# Un-gating and allosteric modulation of a pentameric ligand-gated ion channel captured by molecular dynamics

**DOI:** 10.1371/journal.pcbi.1005784

**Published:** 2017-10-25

**Authors:** Nicolas E Martin, Siddharth Malik, Nicolas Calimet, Jean-Pierre Changeux, Marco Cecchini

**Affiliations:** 1 Laboratoire d’Ingénierie des Fonctions Moléculaire, ISIS, UMR 7006 CNRS, Université de Strasbourg, Strasbourg, France; 2 CNRS, URA 2182, F-75015 & Collège de France, Paris, France; University of Maryland School of Pharmacy, UNITED STATES

## Abstract

Pentameric ligand-gated ion channels (pLGICs) mediate intercellular communication at synapses through the opening of an ion pore in response to the binding of a neurotransmitter. Despite the increasing availability of high-resolution structures of pLGICs, a detailed understanding of the functional isomerization from closed to open (gating) and back is currently missing. Here, we provide the first atomistic description of the transition from open to closed (un-gating) in the glutamate-gated chloride channel (GluCl) from *Caenorhabditis Elegans*. Starting with the active-state structure solved in complex with the neurotransmitter L-glutamate and the positive allosteric modulator (PAM) ivermectin, we analyze the spontaneous relaxation of the channel upon removal of ivermectin by explicit solvent/membrane Molecular Dynamics (MD) simulations. The *μ*s-long trajectories support the conclusion that ion-channel deactivation is mediated by two distinct quaternary transitions, i.e. a global receptor twisting followed by the radial expansion (or blooming) of the extracellular domain. At variance with previous models, we show that pore closing is exclusively regulated by the global twisting, which controls the position of the *β*1-*β*2 loop relative to the M2-M3 loop at the EC/TM domain interface. Additional simulations with L-glutamate restrained to the crystallographic binding mode and ivermectin removed indicate that the same twisting isomerization is regulated by agonist binding at the orthosteric site. These results provide a structural model for gating in pLGICs and suggest a plausible mechanism for the pharmacological action of PAMs in this neurotransmitter receptor family. The simulated un-gating converges to the X-ray structure of GluCl resting state both globally and locally, demonstrating the predictive character of state-of-art MD simulations.

## Author summary

Pentameric ligand-gated ion channels (pLGICs) control membrane conductance in living systems from bacteria to humans. Here, we report on *μ*s-long, atomistic Molecular Dynamics simulations of the glutamate-gated chloride channel (GluCl) with an explicit treatment of the solvent and the membrane environment. The calculated trajectories provide a complete and time-resolved visualization of ion-channel deactivation in pLGICs. Strikingly, two independent simulations started with an open channel stabilized by agonist binding (i.e. both L-glutamate and ivermectin) converge to the X-ray structure of GluCl in the absence of ligands upon removal of the positive allosteric modulator ivermectin. These simulations visualize the gating isomerization in pLGICs with unprecedented space and time resolution and provide a plausible mechanism for the pharmacological action of positive allosteric modulators (PAMs). Our analysis demonstrates in the clearest fashion the predictive power of state-of-art computer simulations and opens to the rational design of drugs targeting pLGICs’ dysfunction including Alzheimer’s, Parkinson’s, depression, or nicotine addiction.

## Introduction

Pentameric ligand-gated ion channels (pLGICs) play a central role in the intercellular communication in the brain and are involved in fundamental processes such as learning, attention, and memory [1]. They are membrane-bound oligomeric proteins that convert a chemical signal, typically the local increase in the concentration of neurotransmitter, into an ion flux through the post-synaptic membrane [2]. At rest, the ion channel is closed and binding of the neurotransmitter to the extracellular (EC) domain elicits a fast isomerization, which results into the opening of a transmembrane (TM) pore and a corresponding flux of cations (or anions) that diffuse at rates approaching tens of millions of ions per second. This process is commonly referred to as “gating” [3].

Prominent members of the pLGIC family in humans include excitatory receptors like the nicotinic acetylcholine receptor (nAChR), which are associated with a cationic channel, and inhibitory receptors like the GABA_*A*_ receptor, which are linked with an anionic channel. Signal transduction by pLGICs is allosterically regulated by ligand binding at sites that are topographically distinct from the neurotransmitter-binding or orthosteric site. The design of small molecules able to activate (agonists), inhibit (antagonists), or modulate (positive or negative allosteric modulators) the function of pLGICs is critical for the development of pharmacological strategies against a range of neurological disorders including Alzheimer’s, Parkinson’s, schizophrenia, and depression. [4]

**Fig 1:**
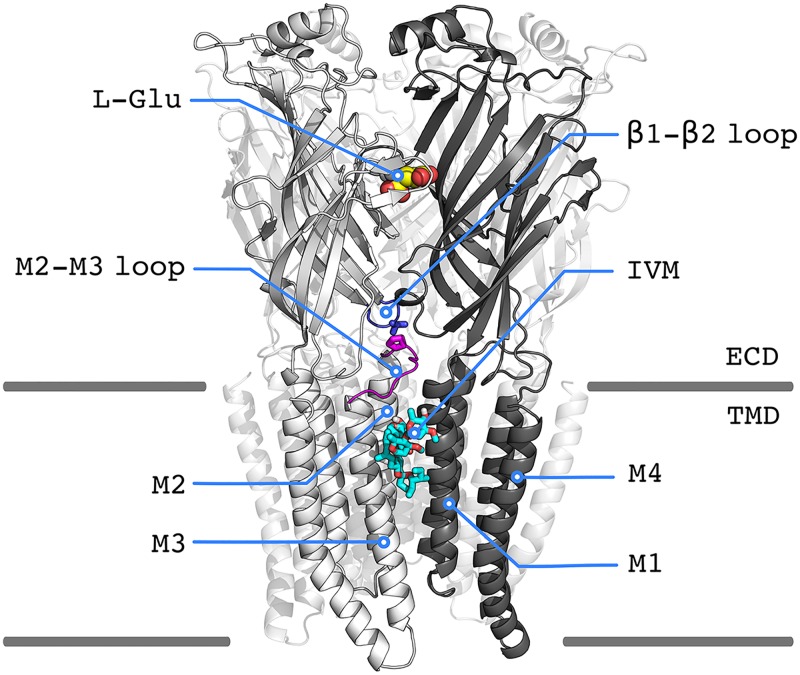
Cartoon representation of GluCl active with L-Glutamate (L-Glu) and ivermectin (IVM) bound; PDB 3RIF. Two out of the five chains are represented in light and dark grey, respectively. The lipid membrane is materialized by grey lines so as to visualize the structural regions corresponding to the extracellular (ECD) and the transmembrane (TMD) domains. The interfacial loops *β*1-*β*2 (ECD) and M2-M3 (TMD) are shown in blue and magenta colors, respectively. The four transmembrane helices per subunit (M1 to M4) are indicated.

Despite the fact that pLGICs and related dysfunction have attracted significant pharmacological interest, the molecular mechanism of signal transduction remains to be elucidated. X-ray crystallography of prokaryotic homologues identified in *Gloeobacter violaceus* (GLIC) [5] and *Erwinia chrysanthemi* (ELIC) [6] provided the first high-resolution descriptions of the open and closed states of the channel. X-ray structures of the eukaryotic glutamate-gated chloride channel (GluCl) from *Caenorhabditis elegans*, which was solved in complex with the endogenous neurotransmitter L-glutamate (L-Glu) and the positive allosteric modulator ivermectin (IVM) [7], and later with phospholipids [8], have provided detailed information on the interaction with a variety of modulatory ligands; see Fig 1. And, high-resolution structures of GLIC at pH7 [9] and GluCl apo [8], which both captured a pLGIC in the absence of agonist, demonstrated that ion gating is mediated by a large conformational change of the receptor, which involves both global twisting as originally proposed based on modeling [10] and the radial expansion or “blooming” of the EC domain. Finally, the most recent structural determinations of the GABA_A_ receptor [11], the 5-HT_3_ receptor [12], the Gly receptor [13, 14], and the *α*_4_*β*_2_ nicotinic receptor [15] started to illuminate the details of the signal transduction mechanism: (i) visualizing a state that is most consistent with desensitization [11]; (ii) providing an atomistic description of the regulatory intracellular (IC) domain [12]; and shedding light onto pLGICs activation/deactivation by agonist versus antagonist binding [13, 14].

The significant amount of structural information available in pLGICs provides opportunities to explore gating by all-atom Molecular Dynamics (MD) [16]. By analyzing the spontaneous relaxation of the open-channel structure triggered by the removal of agonist, MD simulations of GLIC [17] and GluCl [18] consistently pointed to the existence of an indirect coupling between twisting and blooming, suggesting a sequence of events linking (allosterically) neurotransmitter unbinding to pore closing [18]. Similarly, starting with the X-ray structure of GluCl with IVM and no L-Glu bound [19] or the mouse 5-HT_3_R in the absence of nanobodies [20], the transition to a “water-conducting” channel was captured by introducing the neurotransmitter (L-Glu and serotonin, respectively) at the orthosteric site and relaxing the complex by *μ*s-long MD. Although in both cases the physiological significance of the starting structures remains unclear, i.e. it does not correspond to the resting state visualized by GLIC at pH7 [9] or GluCl apo [8], these studies evidenced a striking correlation between orthosteric agonist binding and pore opening. In addition, the analysis of 5-HT_3_R suggested that rotameric transitions of the pore-lining residues at positions 9′ and 13′, which significantly enlarge the ion-pore diameter with minimal variation of the protein backbone, is key to stabilize an ion-conducting state [20].

Here, we report on the first atomistic description of the functional isomerization from active to rest (un-gating) in a pLGICs. Starting with the open-channel structure of GluCl in complex with L-Glu and the positive allosteric modulator IVM, we analyze its structural relaxation upon removal of the IVM by *μ*s-long MD simulations in the native lipid-membrane environment. The calculated trajectories illuminate the spontaneous isomerization to a closed-channel form that is strikingly similar to the X-ray structure of GluCl apo, thus bridging two physiological states of the channel at atomic resolution. Analysis of two independent realizations of un-gating unveils a novel mechanism of pore closing and illustrates how agonist unbinding from the orthosteric site and/or the allosteric transmembrane site regulates the transition to a non-conductive state. These results provide fundamental insights onto the allosteric mechanism and regulation of pLGICs, offering a plausible interpretation of the pharmacological action of positive allosteric modulators (PAMs).

## Results

The conformational dynamics of GluCl at physiologically relevant conditions, i.e. with the pLGIC embedded in a native lipid membrane and exposed to a physiological concentration of sodium and chloride ions, was investigated by all-atom MD starting with three distinct configurations of the channel corresponding to the X-ray structures of the active state with and without the positive allosteric modulator IVM [7], and GluCl apo [8], which is thought to represent the resting state; see S1 Table. Within this set, two independent 2.5 *μ*s simulations of the active state with IVM removed were used to explore the allosteric mechanism of pore closing; they are referred to as “w/o IVM” run A and run B throughout the Text. The simulations of the active state with IVM and L-Glu bound (470 ns) and the resting state (180 ns) with no ligand bound are referred to as “with IVM” and “apo”, respectively. Finally, the simulation of GluCl active with IVM removed and L-Glu restrained to the crystallographic binding mode (290 ns) is referred to “L-Glu*”. In the *Results* section, the description of the spontaneous isomerization from open to closed is presented first. Its mechanistic interpretation follows. The section closes with an analysis of the modulation by ligand binding events.

### Spontaneous transition from active to rest

#### Global structural change

**Fig 2:**
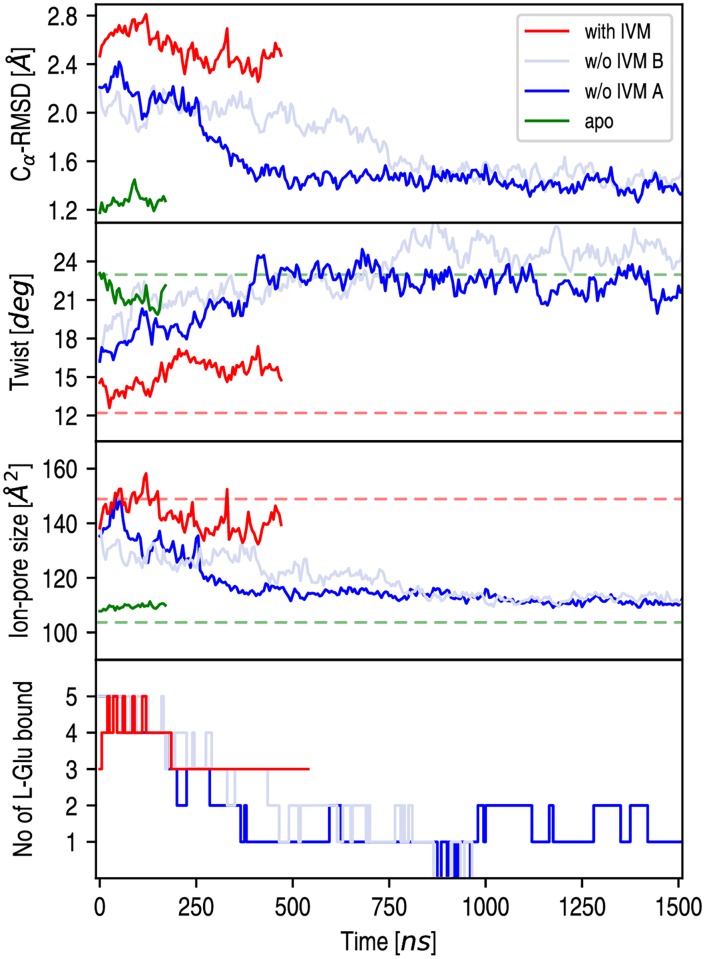
Quaternary change of GluCl active upon removal of ivermectin (IVM). From top to bottom, the time series of the C_*α*_-RMSD of the TMD from the X-ray structure of GluCl apo; the receptor twisting angle; the C_*α*_ cross section of the ion pore at the position 9′; and the number of L-Glu bound are shown. All data points correspond to running averages taken over consecutive time windows of 5 ns (i.e. 500 snapshots). To ease the visualization of spontaneous pore-closing only the first 1.5 *μ*s is shown; full-range analyses are shown in S1 Fig. Red and green dashed lines correspond to values obtained from the X-ray structures of GluCl active (PDB code 3RIF) and rest (PDB code 4TNV), respectively.

The structural evolution of GluCl active with IVM removed was analyzed by monitoring the C_*α*_-RMSD from the resting-state structure (GluCl apo) along the simulated trajectory, which provides a natural progress variable to explore ion-channel deactivation. As shown by Fig 2 (top), the C_*α*_-RMSD of the TM domain starts at 2.2 Å, it decreases over time in both runs with IVM removed and stabilizes to a value of 1.4 Å after 400 ns in run A and 800 ns in run B. Consistently, a converged C_*α*_-RMSD of 1.2 Å for the TM domain was measured from the room-temperature simulation of GluCl apo (Fig 2). In sharp contrast, the conformation of the EC domain differs substantially in the two runs (S2 Fig) mostly as a result of an additional quaternary reorganization in run A, as we shall see.

The relaxation of the active state with IVM removed was further analyzed by monitoring the receptor twisting angle and the size of the ion pore at the constriction point (position 9′), which are both compared with values measured in the active- (with IVM) and the resting-state (apo) simulations in Fig 2 (middle panel). The results show that the removal of IVM corresponds to a global twisting transition in both MD runs, with the twisting angle increasing from 12.2° (red dashed line) characteristic of the active state, to 23° (green dashed line) consistent with the resting state. The degree of pore opening, which was analyzed using a geometric measure based on the Cartesian coordinates of the C_*α*_ atoms at position 9′ (see Methods), indicates that upon removal of IVM the C_*α*_-cross section shrinks from 1.4 to 1.15 nm^2^ in less than 1 *μ*s approaching values that are consistent with the X-ray structure of GluCl apo and corresponding MD (Fig 2). In sharp contrast, when IVM is bound the C_*α*_-cross section fluctuates around a value of 1.4 nm^2^ consistent with the X-ray structure of the active state.

Taken together, these results indicate that in the absence of IVM the active state of GluCl is conformationally strained and relaxes to a globally twisted, closed-channel form that closely resembles the X-ray structure of GluCl apo in the TM domain, but differs considerably in the EC domain. Importantly, they also indicate that the quaternary reorganization captured by MD is independent of the initial simulation conditions, although its spontaneous relaxation may be sampled on substantially different time spans, i.e. 400 ns in run A and 800 ns in run B; confront the light-blue versus dark-blue traces in Fig 2.

#### Pore closing

**Fig 3:**
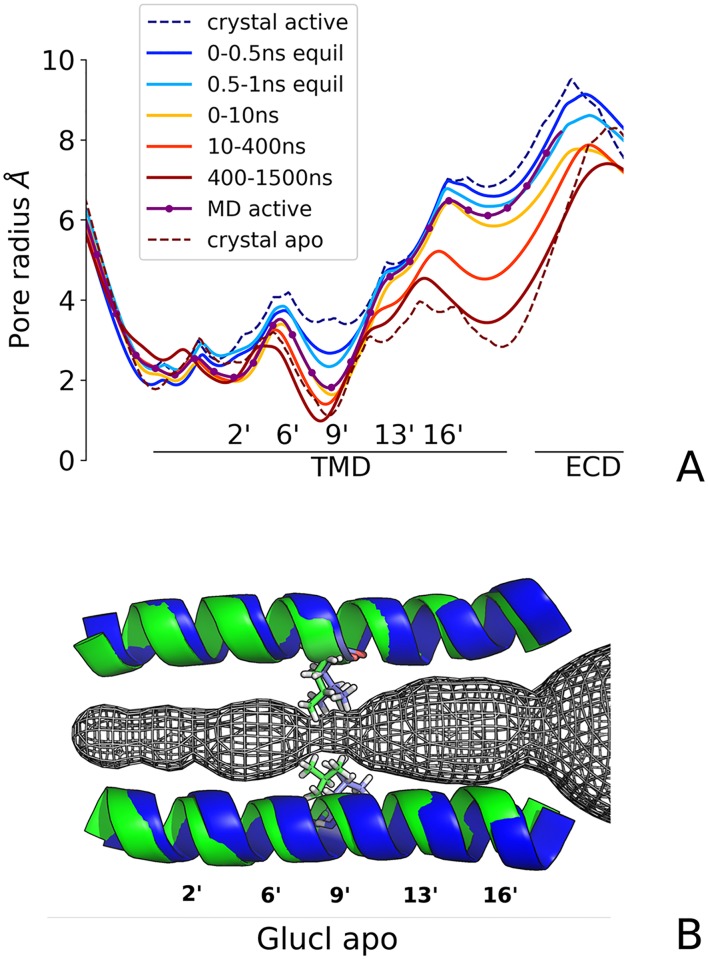
The pore-closing transition of GluCl active promoted by the removal of IVM. (A) The structural evolution of the ion pore is shown by a series of HOLE profiles computed at different time frames. Clearly, the constriction point is located at the position 9′ (Leu 254). (B) The configuration of the pore at the end of the 2.5 *μ*s relaxation with IVM removed. The comparison with the X-ray structure of GluCl apo (green) shows a striking correlation with the MD relaxed structure in the absence of IVM (blue).

To obtain further insight onto the closing isomerization, the radius of the transmembrane pore was monitored during the open to closed simulated transition. For this purpose, a series of snapshots of the protein taken every 0.2 ps were processed by the program HOLE [21]. The resulting profiles averaged over 2-ps windows are shown in Fig 3. The time-resolved analysis indicates that at the end of the relaxation the constriction point is located at the position 9′, fully consistent with the X-ray structure of GluCl apo. Also, it shows that although 50% of closing occurs within the first 2 ns of simulation, where the pore radius at 9′ drops from 3.4 Å (X-ray active) to 1.9 Å (MD with IVM bound), the rest of the transition takes several hundreds of ns. Strikingly, the superimposition of the pore-lining helices M2 on the X-ray structure of GluCl apo shows that in the absence of IVM the TM domain has relaxed to the resting state in less than 1 *μ*s; see Fig 3. Moreover, since the pore radius at position −2′ remains open, i.e. it stabilizes at 1.8 Å versus 1.5 Å in the X-ray structure of the GABA_*A*_ receptor in complex with benzamidine [11] and 1.0 Å in GlyR [22], the pore radius at the desensitization gate (at position -2′) remains open, we conclude that the closed-channel structure sampled by MD at the end of the relaxation with IVM removed corresponds to the resting rather than the desensitized state.

**Fig 4:**
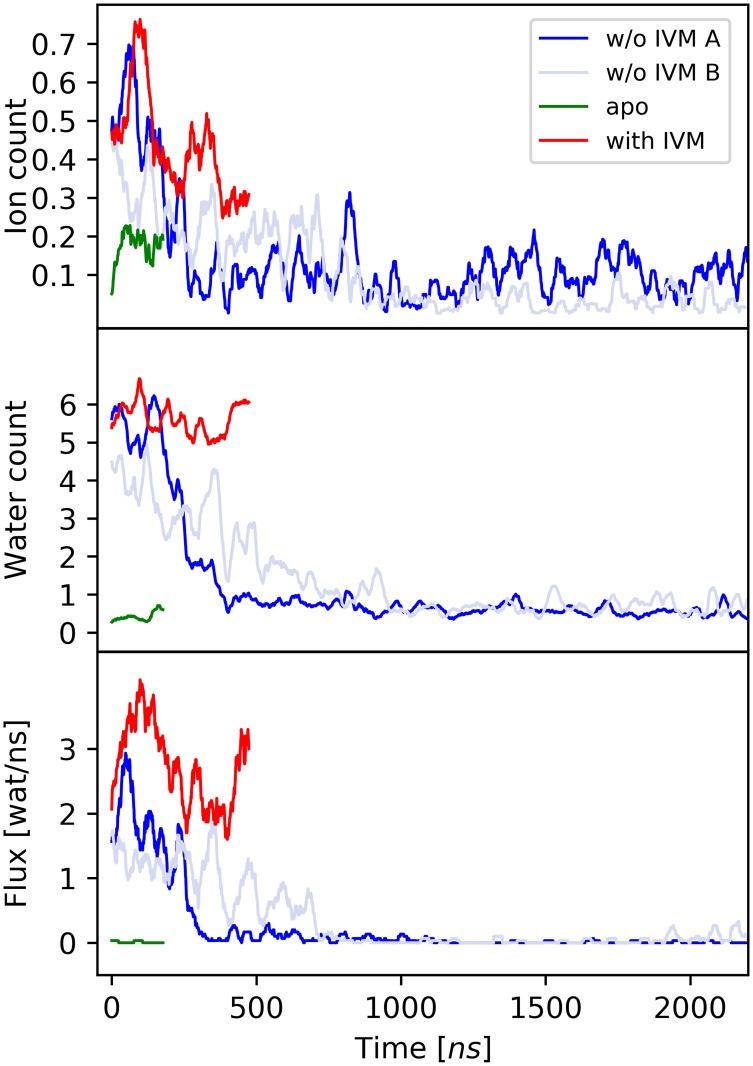
Ion and water permeability in the four simulations of GluCl. On top and middle panels, the number of water molecules and chloride ions sitting in the pore region per nanosecond is monitored over time. On bottom, the time series of the water flux through the pore in shown. Strikingly, in the simulation of GluCl with IVM removed (dark and light blue) the number of water molecules inside the pore drops from six to nearly zero in 400 ns in run A, and 800 ns in run B. In sharp contrast, when IVM is bound (red) the average number of water molecules in the pore fluctuates around six.

To explore channel permeability in the various conformational states of GluCl, the time evolution of the water/ion distribution around the pore and the water/ion permeation or flux through the pore were analyzed; see Methods. These observables provide an orthogonal and non-structural definition of channel closing [23]. The results in Fig 4 (bottom) show that in the presence of IVM the ion-channel is water permeable with an average flux of 1.9 water per nanosecond. In sharp contrast, upon removal of IVM the number of pore-wetting waters close to the constriction point goes to zero in 400 ns (run A) or 800 ns (run B), with the flux stabilizing at 0.01 water/ns at the end of the relaxation. Perhaps surprisingly, the results show that chloride ions never cross the constriction point even when IVM is bound, although they visit the transmembrane pore region with an average count of 0.5 ion/ns (Fig 4). The lack of an ionic concentration gradient and/or the absence of a transmembrane potential in the simulations might explain this surprising observation. Upon pore shutting (400 ns run A, or 800 ns run B), the ion count drops to 0.1 ion/ns consistent with the simulation of GluCl apo. Therefore, the structural relaxation of GluCl upon removal of IVM produces a significant shrinking of the transmembrane pore, mostly as a consequence of the inward displacement of the bulky and hydrophobic residue Leu 254 (Fig 3B), which eventually results in partial pore dewetting. Analysis of the water distribution inside the pore indicates that the dehydrated stretch upon closing is 4.8 and 4.6 Å long for run A and B respectively (see S17 Fig). In addition, the striking correlation between the position of the dehydrated stretch and the water-occluded region evidenced by HOLE (S17 Fig) suggests that pore-closing in these simulations is more consistent with physical constriction than hydrophobic gating.

Taken together, the simulation results indicate that at physiologically relevant conditions GluCl with IVM bound is a water-permeable channel featuring a transmembrane pore that is narrower than the one observed in the crystals. The removal of IVM triggers a rapid (sub-microsecond) isomerization of the TM domain that shuts the ion pore at position 9′, consistent with the crystallographic result in the absence of ligands (Fig 3B). We conclude that both *μ*s simulations of GluCl active with IVM removed have captured the full closing transition, thus providing a time-resolved description of ion-channel deactivation or un-gating in pLGICs with atomic resolution.

### The allosteric mechanism of pore closing

The two *μ*s relaxations of GluCl active with IVM removed were analyzed in greater detail to collect insights onto the allosteric mechanism that couples orthosteric agonist binding to pore closing almost 60Å away.

#### The orthosteric site

By monitoring the position of the five L-Glu at the orthosteric site, we found that L-Glu unbinds spontaneously during the relaxation of GluCl with IVM removed; see Fig 2 (bottom). Intriguingly, the results indicate that the release of the fourth (out of five) L-Glu from the orthosteric site is time-correlated with receptor’s twisting and pore closing in both MD runs; i.e. at 400 ns in run A and 800 ns in run B. These observations suggest that global twisting is regulated by agonist binding at the orthosteric site, i.e. complete twisting does not occur as long as two L-Glu are bound. Also, they indicate that in the absence of IVM L-Glu alone is not sufficient to stabilize the active state of GluCl in free MD.

#### The extracellular domain

**Fig 5:**
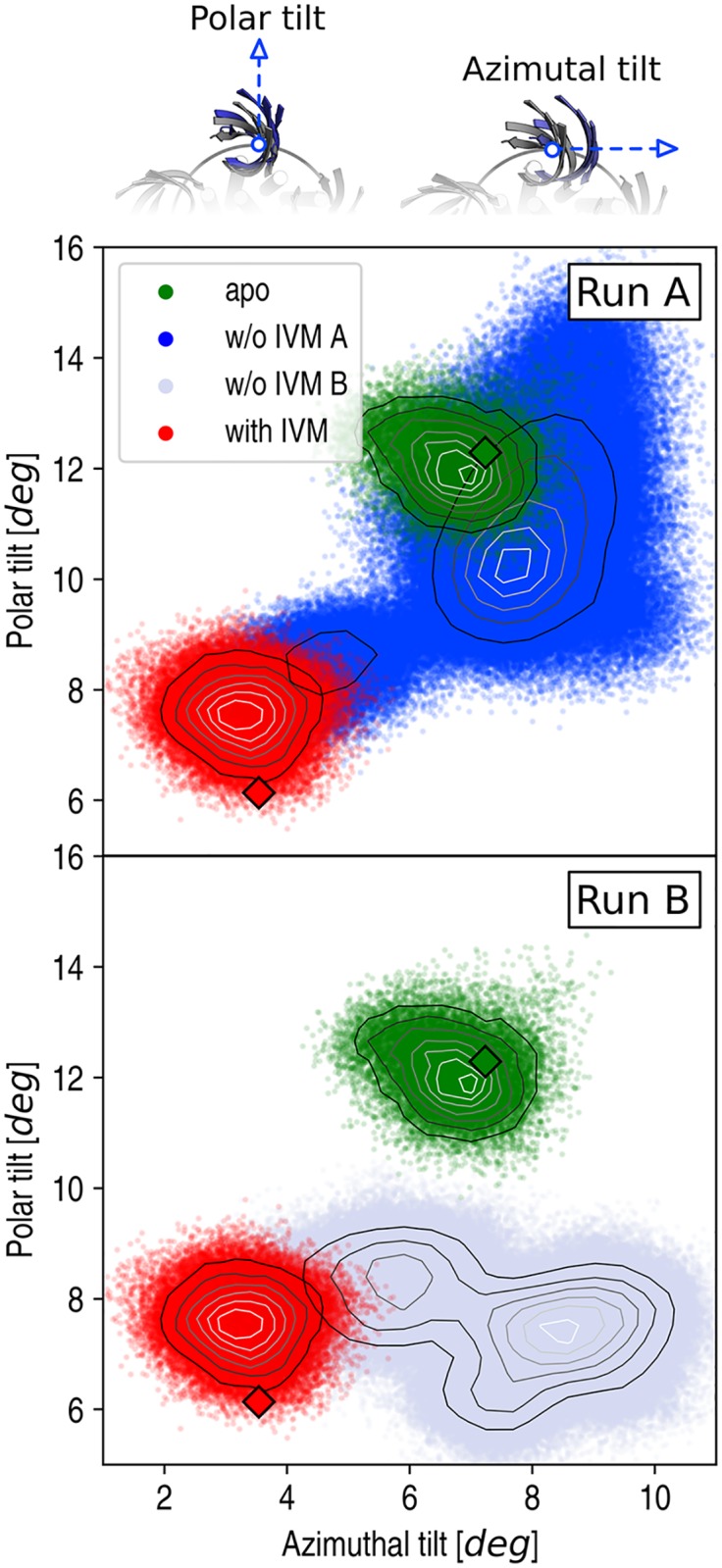
The conformational dynamics of the EC domain. The polar (*θ*_*p*_) versus azimuthal (*θ*_*a*_) components of the tilting angle of the EC subunits are plotted along the MD simulations of GluCl active (red), resting (green), and the transition from active to rest (blue). Red and green diamonds correspond to values measured in the X-ray structures of GluCl. Isocontour lines show the density of points, i.e. the higher the density, the lighter the color and are used to highlight the existence of marginally stable states sampled by MD during the relaxation with IVM removed. On top, the polar and azimuthal tilting components are illustrated using snapshots extracted from the simulations.

The quaternary reorganization of the EC domain was investigated by monitoring the orientation of the *β*-sandwiches, whose radial and tangential tilting were analyzed over time; see Ref. [18] for the definition of the corresponding polar and azimuthal tilting angles. The results in Fig 5 show that in the active state (red) the EC domain is significantly more contracted (Δ*θ*_*p*_ of −4.5°) and straight (Δ*θ*_*a*_ of −3.4°) than the one in the resting state (green). Also, they show that the removal of IVM (dark and light blue) results into a tangential tilting of the *β*-sandwiches (Δ*θ*_*a*_ of 5° for run A and 6° for run B), which appears to be coupled to the radial expansion of the EC domain in run A (Δ*θ*_*p*_ of 2.4°) but not in run B (Δ*θ*_*p*_ of 0.2°); confront the dark-blue and light-blue clouds in Fig 5. Because pore closing occurs in both runs with IVM removed (see above), these results indicate that tangential rather than radial tilting of the EC subunits is a molecular requirement for closing. Also, since tangential tilting is strongly correlated with receptor twisting (S8 Fig), this analysis suggests that the twisting isomerization controls channel closing.

The results in Fig 5 highlight the occurrence of a second isomerization of the ion channel, which was captured by run A but not run B. This additional conformational change corresponds to the radial expansion or *blooming* of the EC domain, which results from a global reorientation of extracellular subunits in the radial direction; the average polar tilting angle increases from 7.6° to 10° in run A. Strikingly, the significant overlap between the green (GluCl apo) and dark blue (GluCl w/o IVM) clouds in Fig 5 indicates that the blooming is required to reach the physiological resting state. Also, the large-amplitude fluctuations sampled by the EC subunits at the end of the MD relaxation show that the EC domain is significantly more flexible in the (bloomed) resting state than the (unbloomed) active state, consistent with previous observations on GLIC [9]. Visual inspection of the simulated trajectory reveals that the enhanced conformational variability in the EC domain upon blooming originates from a remarkable loss of coupling between the subunits, which become free to reorient almost independently (see S1 Video). The corresponding loss of pentameric symmetry explains the larger structural deviation of the EC domain observed in both run A and the room-temperature simulation of GluCl apo relative to the X-ray structure of the resting state (S2 Fig). Strikingly, these results show that the MD relaxation of GluCl with IVM removed in run A bridges the gap from active to resting through a complex conformational change that involves the sequential activation of two quaternary isomerizations, i.e. global twisting followed by blooming of the EC domain.

#### The EC/TM domains interface

**Fig 6:**
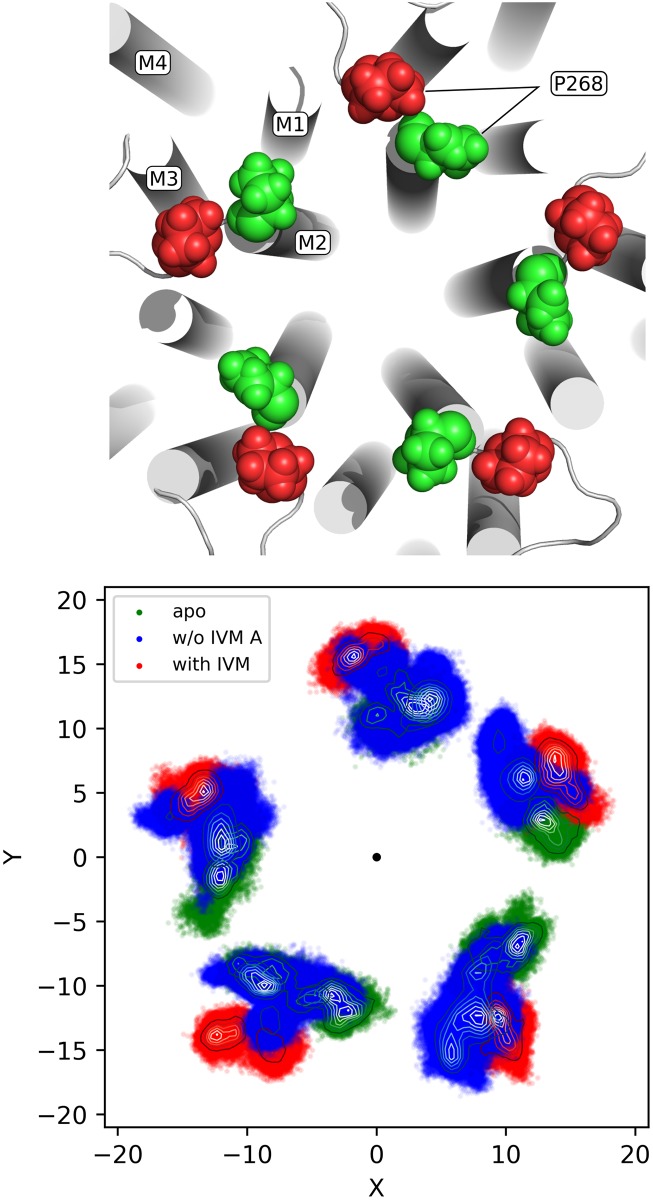
Structural rearrangement at the EC/TM domains interface during ion-channel deactivation. On top, the location of the strictly conserved proline (P268) at the EC/TM interface is shown. The four transmembrane helices and the position of P268 in the active (red) and resting (green) states are indicated. On bottom, the spatial distribution of the center of mass of the five P268 on the plane parallel to the membrane is shown for GluCl active with (red) and without IVM (blue), and GluCl resting (green). The center of the pore is represented by a large black dot.

The rearrangement of the *β*1-*β*2 loop and the M2-M3 loop at the EC/TM domains interface was analyzed during the pore-closing transition. Earlier simulations of GluCl suggested that a horizontal displacement of the M2-M3 loop in the direction of the ion pore is crucial for gating [18]. To substantiate this conclusion, the position of the totally conserved proline on the M2-M3 loop [24] (P268 in GluCl) was monitored by projecting its center of mass on a plane perpendicular to the principal axis of the receptor upon superimposition of the TM domain. The results in Fig 6 show that with IVM bound (red) these interfacial prolines adopt an *out* position, which is distinct and non-overlapping with the *in* position characteristic of the resting state (green). However, upon removal of IVM (blue), all prolines move from *out* to *in* by approximately 4 Å (S7 Fig), ultimately populating spatial distributions that strongly overlap with those sampled by the simulation of GluCl apo (green). Similar results were obtained for run B (see S3 Fig). Consistent with previous observations [18, 25], these results support the conclusion that a fully-closed configuration of the channel (characteristic of the resting state) is reached through the inward displacement of the M2-M3 loop at the EC/TM domains interface. Since the translocation of this loop involves the passage of the bulky proline 268 past the tip of the *β*1-*β*2 loop [18], they also indicate that the position of the *β*1-*β*2 loop at the EC/TM interface is crucial to regulate pore closing.

#### The transmembrane domain

The structural/functional consequences of the translocation of the M2-M3 loop at the EC/TM interface were analyzed by measuring the correlation between the position of the interfacial P268 and the cross section of the ion-pore in the TM domain. The strong correlation in S9 Fig indicates that the position of this proline controls pore closing 20 Å away, i.e. allosterically.

To assess whether pore closing involves the decoupling of the M2 helix from M3 within one subunit in the TM domain, as proposed for GLIC [9, 25], the separation between the geometrical centers of residues 258 to 265 (on M2) and 274 to 280 (on M3) was monitored along the open to closed simulated transition. The results indicate that despite significant shrinking of the pore the separation between these two transmembrane helices is stable over time with an average value of 12.2 Å (S5 Fig). Also, the corresponding distances in the X-ray structures of the locally-closed (LC) state of GLIC [25] (14.2 Å using 4NPP) or GLIC pH7 (14.04 Å using 4NPQ) are clearly non overlapping with the fluctuations sampled by MD of GluCl with IVM removed (S5 Fig). We conclude that pore shutting in GluCl is the result of a highly coordinated movement of the M2 and M3 helices with no need for uncoupling. These results along with the X-ray structure of GluCl apo [8] challenge the significance of the closed-channel structures of GLIC [9, 25] and suggest either a unique pore-closing mechanism in this prokaryotic family, or an alternative functional annotation for these structures.

#### Allosteric coupling

**Fig 7:**
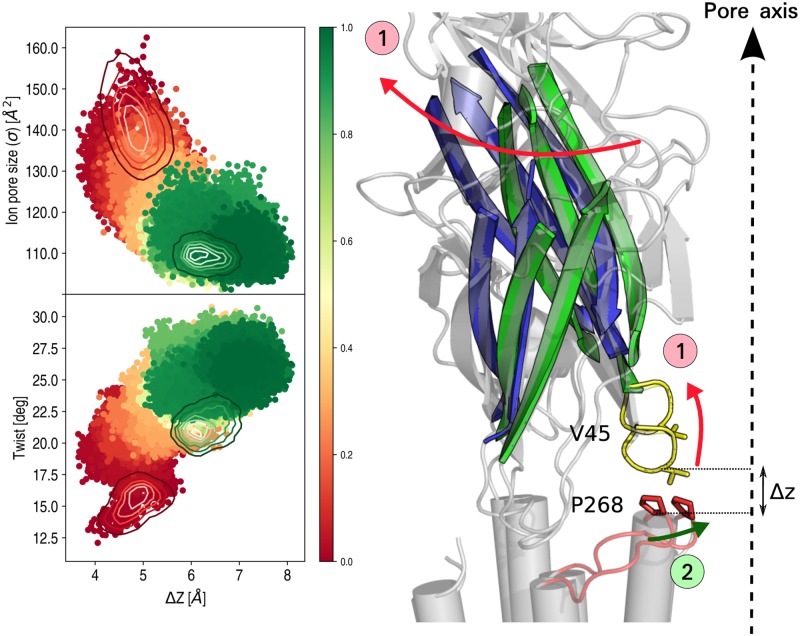
An upward movement of the *β*1-*β*2 loop correlated with the twisting isomerization couples orthosteric agonist unbinding to pore closing in GluCl. On the left, the correlation between the vertical separation of the *β*1-*β*2 loop from the M2-M3 loop (Δ*Z*) with the twisting angle (*τ*) and the cross section of the pore at the constriction point are shown. Δ*Z* is computed as the distance, projected on the Z axis, between the *α*-carbons of residues P268 (on M2-M3 loop) and V45 (on *β*1-*β*2 loop) averaged over the 5 subunits. The isocontour lines correspond to the simulations of GluCl active (red) and resting (green). The color gradient from red to green illustrates the time evolution of GluCl active with IVM removed. On the right, the gating mechanism is illustrated using snapshots taken at the beginning (red) and the end (blue) of the MD relaxation. Upon L-Glu unbinding, global receptor twisting results in an upward movement of the *β*1-*β*2 loop that facilitates the passage of the bulky proline 268 at the EC/TM interface to shut the pore at position 9′.

The analysis above supports the conclusion that receptor’s twisting is the only molecular requirement for channel closing in GluCl. Also and consistent with previous observations [18, 25], it indicates that the position of *β*1-*β*2 loop at the EC-TM interface controls pore closing through its interaction with the totally conserved proline on the M2-M3 loop. If so, a complete model of closing would require an understanding of the coupling between twisting and the relative position of the EC/TM interfacial loops. To this aim, the displacement of the *β*1-*β*2 loop from the M2-M3 loop was analyzed during the *μ*s relaxation of GluCl with IVM removed by monitoring the vertical separation between the C_*α*_ carbons of P268 (on the M2-M3 loop) and V45 (at the tip of the *β*1-*β*2 loop), here termed Δ*Z*. The results indicate that Δ*Z* increases from 5 Å characteristic of the active state, to 7 Å consistent with the simulation of GluCl apo (Fig 7). Also, they show that the vertical displacement of the *β*1-*β*2 loop is strongly correlated with the degree of twisting and anticorrelated with the size of the ion pore; i.e. a distance increase of 2 Å along the vertical direction corresponds to pore-shrinking that is consistent with the closed-channel state (e.g. GluCl apo). Interestingly, visual inspection of the EC/TM interfacial region before and after pore closing unveils that the tangential reorientation of the EC subunits during the twisting transition produces a striking upward movement of the *β*1-*β*2 loop, which facilitates the inward displacement of the P268 residue at the EC/TM interface; see Fig 7. Taken together, these results indicate that the local rearrangement required for closing is a vertical displacement of the *β*1-*β*2 loop at the EC/TM interface, whose position is controlled by the global twisting of the receptor. Since global twisting may be regulated by agonist binding at the orthosteric (L-Glu) and/or the allosteric transmembrane (IVM) sites (see above), this analysis indicates that *quaternary* twisting alone provides an allosteric coupling for gating. Also, it suggests that the totally conserved proline on the M2-M3 loop (P268) would act as a sensor for agonist binding thorough its interaction with the *β*1-*β*2 loop.

### Modulation by orthosteric versus allosteric ligand-binding events

**Fig 8:**
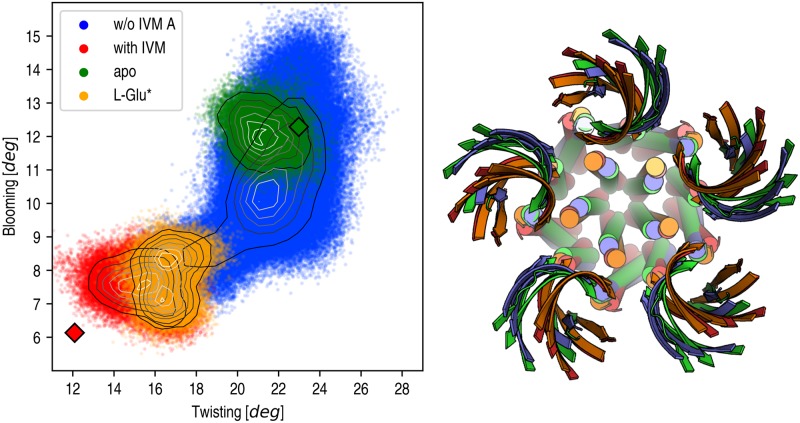
Receptor twisting versus blooming for the four simulations of GluCl. The simulations of the end points of gating in green (resting) and red (active) sample values of the twisting and blooming reaction coordinates that are consistent with the X-ray structures of GluCl apo (green diamond) and GluCl with IVM bound (red diamond), respectively. When IVM is removed, a striking evolution of both twisting (from 15° to 22°) and blooming (from 8° to 10°) angles is observed. When only L-Glu is bound (orange) the receptor is partially twisted (*τ* of 17°) and contracted (*θ*_*p*_ of 8°). Isocontour lines on the relaxation of GluCl with IVM removed (blue) are used as visual guidelines to show the existence of a kinetic intermediate sampled in the early stages of the simulation. On the right-hand side, representative structures for the four simulations are shown using the same color code. The structural comparison illustrate the global character of the gating isomerization.

The MD relaxation of GluCl promoted by the removal of IVM showed that ion-channel deactivation is mediated by two sequential isomerizations. By projecting the 2.5*μ*s relaxation (run A) on the twisting and blooming (polar tilting) reaction coordinates an interesting scenario emerges (Fig 8). The results show that when IVM and L-Glu are both bound (red), the receptor is stable in a globally untwisted or *straight* configuration with a contracted EC domain; the twisting and blooming angles fluctuate around average values of 15.3° and 7.6°, respectively. On the other hand, when no agonist is bound (green), the receptor adopts a globally twisted configuration with a radially expanded EC domain; corresponding twisting and blooming angles are 21.3° and 12.1°, respectively. Interestingly, when IVM is removed (blue), the receptor does not reach the resting state immediately. Rather, it evolves to an intermediate configuration that is significantly more twisted (*τ* ≈ 18°) but still preserves a contracted EC domain (*θ*_*p*_ ≈ 8°). The kinetic stability of this marginally stable state is not negligible (i.e. 400 ns) and is time-correlated with the presence of L-glutamate (Fig 2, bottom), whose binding to the orthosteric site hinders the full twisting isomerization (see above).

To explore the significance of this kinetic intermediate sampled by MD on closing, an additional simulation of GluCl active with IVM removed was carried out by introducing harmonic restraints on the L-Glu/receptor interactions to stabilize neurotransmitter binding; see Methods. The L-Glu restrained simulation (L-Glu*) shows that orthosteric agonist binding alone stabilizes a unique quaternary organization of the pentamer, which is more twisted (*τ* of 16.7°) than the one stabilized by IVM binding (*τ* of 15.3°) and closely resembles the kinetic intermediate captured by un-restrained MD on closing; see Fig 8. To collect additional information, the configuration of the orthosteric site, the allosteric site and the ion pore in the L-Glu* simulation were compared with those observed in the resting-state and active-state simulations. To this aim, the separation between the C_*α*_ atoms of Ser150 (+) from Arg56 (-) in the EC domain and G281 (+) from L218 (-) in the TM domain were monitored over time; these residues sit at the subunits interface and form strong interactions with either L-Glu or IVM in the X-ray structure of the active state [1]. The results (S2 Table) show that in the L-Glu* simulation the orthosteric pocket is active-like and is significantly more contracted than that in the resting state or at the end of the relaxation of GluCl with IVM removed. By contrast, the allosteric site in the TM domain is more resting-like and its cavity shrinks by 1.2 Å relative to active. Finally, analysis of the transmembrane pore by HOLE shows that the radius at the constriction point is 1.81 Å in the L-Glu* simulation, which closely matches the value measured in the active state with IVM bound (1.86 Å). Based on these observations, we conclude that the conformation of GluCl sampled by L-Glu* or in the early stages of the MD relaxation with IVM removed is a quaternary distinct open-channel form featuring a globally more twisted architecture and a closed allosteric site, but that still preserves the exact same open-pore configuration, i.e. it is functionally equivalent to the IVM bound state.

## Discussion

Pentameric ligand-gated ion channels (pLGICs) are transmembrane protein assemblies that mediate interneuron communication by opening an ion pore in response to increased levels of neurotransmitter at synaptic terminals. Despite the recent availability of high-resolution structures from prokaryotes [5, 6, 9, 25], invertebrate [7, 8] and vertebrate [12, 13] eukaryotes including humans [11, 14], a detailed understanding of gating, i.e. the conformational transition leading to an open pore in response to agonist binding, is still missing. Only recently, a two-step asymmetric mechanism has started to emerge [1].

Here, we report on the functional isomerization of the glutamate-gated chloride channel (GluCl) from the active state stabilized by agonist binding at both the orthosteric (L-glutamate) and the allosteric transmembrane (ivermectin) sites to the resting state by microsecond, explicit solvent/membrane Molecular Dynamics (MD). In the spirit of a previous study by us [18], we have analyzed the spontaneous relaxation of the channel upon removal of ivermectin (IVM) and infer on the pore-closing mechanism. Importantly and unlike in previous reports [17, 18], the present simulations capture the full conformational transition to the physiological resting state at an atomic level of detail. Consistent with previous models [18, 26] as well as X-ray crystallography of the end states [7, 8], the removal of IVM results in a closed-channel structure that is globally *twisted* and presents an expanded or *bloomed* configuration of the extracellular (EC) domain. Strikingly, the totally conserved prolines on the M2-M3 loop (P268), which are “out” in the active state, have all moved “in” at the end of the MD relaxation, and the transmembrane pore shuts at the position 9′ (L254), consistent with the X-ray structure of GluCl apo [8]. These results show that MD simulations started from the high-resolution structure of an open GluCl stabilized by ligands [7] were able to produce a closed-channel state that is both globally and locally consistent with the crystallographic result in the absence of ligands [8]. Hence, they suggest that state-of-art Molecular Dynamics may be used in some cases as a predictive tool. In addition, they provide the details of the closing isomerization, which are not directly accessible by experiments, shedding new light onto the gating/un-gating mechanism in pLGICs. The most important findings are summarized below.

First, the simulation results indicate that in the absence of IVM the active state of GluCl is conformationally strained (or *tensed*) and relaxes to a globally twisted, closed-pore form that closely resembles the structure of GluCl apo [8]. Consistent with previous hypotheses [18, 25], they provide evidence that the closed-pore configuration is reached through an inward displacement of the M2-M3 loop at the EC/TM interface, which involves the passage of the bulky proline 268 past the tip of the *β*1-*β*2 loop. Thus, they support the conclusion that opening/closing of the channel is regulated by receptor twisting *indirectly* [18] and that gating/un-gating essentially corresponds to rearranging loops in the crowded environment of the EC/TM interface. However and in disagreement with previous conclusions [18], we demonstrate that the position of the *β*1-*β*2 loop relative to the M2-M3 loop can be entirely controlled by the twisting isomerization, which appears to be the only molecular requirement for closing. In light of this, the present simulations are consistent with a mechanism for closing in which agonist unbinding is the initiating event, a global twisting isomerization follows, which shifts the *β*1-*β*2 loop away from the EC/TM interface, and the horizontal translocation of the M2-M3 loop coupled to an inward untilting of the pore-lining helices M2 completes the transition by shutting the pore at the position 9′. Since the same global twisting transition appears to be regulated by L-Glu or IVM binding at topographically distinct sites, this analysis demonstrates that quaternary twisting alone provides an allosteric coupling for pore opening/closing, as originally proposed based on a model of *α*7 [10].

Second, our MD relaxations of GluCl with IVM removed reveal the existence of a previously unreported metastable state on closing. This “new” intermediate structurally corresponds to an open-channel form that is globally more twisted than GluCl with IVM bound [7] and features a closed allosteric pocket in the TM domain, but that still preserves the exact same open-pore configuration. As its kinetic stability is controlled by L-Glu unbinding, we conclude that neurotransmitter binding in the absence of allosteric modulators would stabilize a quaternary distinct open-channel form, consistent with recent reports on the Gly receptor [13]. Importantly, our analysis makes it clear that agonist binding at the orthosteric (L-Glu) or the allosteric transmembrane (IVM) sites modulate the same twisting isomerization with IVM binding promoting the transition to a *super-untwisted* state, which is expected to enhance the energetic barrier for closing even further. In this view, the distinct quaternary structure captured by MD in the early stages of the relaxation with IVM removed would be consistent with an active state elicited by neurotransmitter binding alone, i.e. in the absence of modulators. Since the coupling between quaternary twisting and pore-closing is indirect (see above), this interpretation offers a molecular understanding of the pharmacological action of positive allosteric modulators (PAMs), which would increase the open-pore probability with no effect on the ion flux by modulating the twisting isomerization.

Last, our *μ*s-long simulations of GluCl indicate that ion-channel deactivation or un-gating is composed of two distinct quaternary transitions, i.e. a global receptor *twisting* and the radial expansion or *blooming* of the EC domain, which are activated in this order to reach the physiological resting state (S2 Video). However, they also suggest that receptor blooming is not strictly required for closing, which naturally questions the functional significance of the second isomerization. Our simulation analysis provides evidence that upon receptor twisting from active, the radial expansion of the EC domain that results in uncoupled EC subunits, is a stochastic event occurring on the *μ*s timescale. Because this transition involves solvent exposure of large contact areas that are buried in the contracted form, the *μ*s barrier for blooming probed by MD, e.g. in run B must be associated with breaking of extensive interactions at the subunit interfaces. On the other hand, the enhanced conformational variability observed in the bloomed EC domain (S1 Video) is likely to introduce a sizeable entropy stabilization, which must prevent the fast reverse transition to the contracted form as evidenced by the second part of the relaxation with IVM removed in run A and the room-temperature simulation of GluCl apo. Interestingly, the significant barriers for blooming/unblooming in a closed-pore receptor would be consistent with the existence of a pre-active intermediate state on gating [27–29]. Since the thermodynamic stability of this intermediate is supposed to be modulated by ligand binding at the orthosteric site [27], the existence of a blooming isomerization with no (direct) consequence on pore opening/closing might be interpreted as a molecular mechanism for agonist selection. If so, agonism in pLGICs would be related to the ability of a given ligand to stabilize the globally twisted, EC-contracted, and pore-closed receptor, which was captured by MD in one of the two runs with IVM removed (run B), versus the resting receptor. Although these conclusions remain speculative at this stage, the simulation results emphasize that pore-closing and un-gating, i.e. the transition to the physiological resting state, are not quite the same thing.

The mechanistic scenario emerging from the simulations of GluCl prompts the comparison with previous models of gating [18–20, 26, 30, 31], which highlights similarities but also differences. Although existing models generally agree on that gating is mediated by a global isomerization involving both twisting and blooming, they do not provide a precise description of the mechanistic role of these movements, nor of their modulation by ligand binding events. Our present analysis illustrates how both twisting and blooming, and not only twisting [19], fundamentally contribute to the functional isomerization to the physiological resting state, showing that un-gating is mediated by the sequential activation of twisting and blooming in this precise order, rather than the opposite [20]. Moreover, our interpretation offers a unifying mechanistic picture, in which the functional consequences of the more local changes at key sites (i.e. the orthosteric site, the pore lumen, the EC/TM interface, etc.) are subjected to the quaternary re-organization of the channel, which provide allosteric control through ligand-binding events at the subunit interfaces. In this framework, our analysis of GluCl suggests that pore closing is rate limited by the global twisting of the receptor rather than rotameric switching at the hydrophobic girdle [20], which we have shown is irrelevant to water permeation in the open-channel form of GluCl stabilized by IVM binding (S14 Fig). Similarly, it highlights that the structural rearrangements promoted by the removal of IVM, which have been described as specific to IVM binding and not related to gating [19, 26], are part of a more complex quaternary mechanism for gating/un-gating, which is more than the mere opening/closing of an ion pore. Remarkably and in agreement with our interpretation, the mechanistic role of the loops at the EC/TM interface for gating/un-gating in pLGICs has been recently recognized by the string method optimization of the gating pathway(s) in GLIC [31]. However, unlike in Ref. [31], no evidence of causality between *β*-sheet expansion and pore closing was found in our simulated un-gating of GluCl (see S15 Fig).

In conclusion, we have reported on the spontaneous and complete pore-closing isomerization of the eukaryotic, pentameric ligand-gated ion channel GluCl as visualized by two independent 2.5 *μ*s-long explicit solvent/membrane MD simulations. The availability of a time-resolved, atomistic description of the conformational transition between two physiological states corresponding to ion-channel deactivation provides new insights on the molecular mechanism and allosteric regulation of gating. These results considerably enrich our understanding of pLGICs function [3] and offer new opportunities to explore ligand modulation in this important family of neurotransmitter receptors.

## Materials and methods

### Preparation of structures

A detailed description of the preparation of the active state model with and without IVM starting with the X-ray structure of GluCl with L-glutamate and ivermectin bound (PDB 3RIF) [7] is given in Ref. [18]. The L-Glu* simulation was started from the model built with IVM bound. Parameters for IVM were assigned using the CGenFF software [32, 33]. An atomistic model of the resting state was prepared starting from the X-ray structure of GluCl apo (PDB 4TNV) [8] and following the same procedure. Three missing residues per subunits (i.e. 103–105), which form a loop inside the lumen of the channel in the EC domain were reconstructed by MODELLER [34] using the X-ray structure of GluCl with IVM bound as a template. Two intra-subunit disulphide bridges between the cysteine residues 130 and 144, and residues 191 and 202 were built in all subunits as done for the active state. The structure of GluCl apo was analyzed by MOLPROBITY [35] and the suggested flips (30 total, S5 Table for a complete list) were introduced before submitting the structure to energy minimization. The protonation state of the ionizable residues at pH 5.5 for consistency with the crystallization conditions [8] was assigned based on Poisson-Boltzmann calculations [36] and the multi-site titration approach [37]. Amino acids predicted in a non standard protonation state are listed in S4 Table.

### Simulation setup

All-atom MD simulations of the GluCl pentamer with five L-Glu ligands bound to the orthosteric pocket were described in Ref. [18]. Here, the initial sub-*μ*s simulations with and without the allosteric modulator ivermectin (IVM) were extended to 470 ns and 2.5*μ*s, respectively. One independent 2.5*μ*s-long simulation with IVM removed was initiated using a longer equilibration scheme (50 ns versus 2 ns) to minimize the influence of the relaxation regime of the membrane environment on the conformational transition of the pLGIC. The MD simulation of GluCl apo in its explicit solvent and membrane environment was set up using the same protocol. The constructed system included one pentameric protein assembly, 42.034 water molecules, 324 POPC lipids, 119 Na^+^, and 119 Cl^−^ ions for a total of 197.056 atoms. The energetics were modeled using the all-atom CHARMM27 force field (i.e. CHARMM22 [38] with CMAP corrections for backbone dihedrals [39]) for the protein and CHARMM36 for the lipids [40]. The modified water model TIP3P [41] and the NaCl parameters from Roux and coworkers [42] were used for the solvent. The simulations were carried out in the isothermal-isobaric (NPT) ensemble using the highly scalable NAMD package [43]. The pressure was maintained constant to 1.01325 bar by the Berendsen barostat [44], the temperature was controlled by the Langevin thermostat [45] at 300 K. A cutoff of 12 Å was used for the electrostatic interactions with a switch at 10 Å. The electrostatics were computed every two steps and the time step used was 2 fs. All covalent bonds involving hydrogen atoms were constrained with the SHAKE algorithm [46]. The simulation cell was allowed to fluctuate anisotropically while keeping a constant ratio between the x and y dimensions, which are parallel to the membrane plane. The molecular system was equilibrated for 2 ns (NPT ensemble, 1 bar, 300 K), while the positional restraints on the heavy atoms of the protein were gradually turned off.

### Structural and dynamic observables

Five observables were used to characterize the functional state of the pLGIC: the global twisting of the receptor, the tilting of the β-sandwiches in the EC domain, the configuration of the ion pore, its ion and water permeability, and the configuration of the orthosteric neurotransmitter site and the allosteric transmembrane site. All observables have been implemented in the program Wordom (version 0.23-rc1 available at https://sourceforge.net/p/wordom/codehg) to allow for efficient analysis of long MD trajectories.

The **global twisting** (*τ*) was evaluated per subunit and defined as the angle spanned by the projections of the geometrical centers of the EC and the TM portions of each subunit on the pseudo-symmetry axis of the receptor. Geometrically, this angle measures the torsion of the EC domain relative the TM domain of the receptor around the pore axis. For the analysis, receptor twisting per snapshot was evaluated by averaging over the twist angle of its five subunits.

The **extracellular tilting** was also evaluated per subunit and decomposed into polar (*θ*_*p*_) and azimuthal (*θ*_*a*_) components. These two angles were measured in the reference frame of each EC subunit with the Z-axis perpendicular to the plane of the membrane and the X-axis pointing outwards along the radial direction. By naming $\vec{v}$ the vector defining the principal axis of the EC subunit, the polar (radial) tilt was measured as the angle between the Z-axis and the projection of $\vec{v}$ on the XZ plane, whereas the azimuthal (tangential) tilt as the angle between the Z-axis and the projection of $\vec{v}$ on the XZ plane. Similar to the twisting angle, the polar and azimuthal tilt used for the analysis correspond to averages over the five subunits per snapshot.

The **opening** of the transmembrane pore was probed by measuring its radius at the constriction point (residue 9′, Leu 254) by HOLE [21] or the C_*α*_ cross section at position 9′ (*σ*) using a simple geometric definition [10]; the latter is referred to as the “ion-pore size” throughout the text. Pore dehydration and ion permeation were probed by counting the number of water molecules or ions within a cutoff distance of 2 and 5 Å, respectively, from the constriction point. The latter provides an orthogonal and not structure-based measure of pore opening. This analysis was done using the toolbox of VMD 1.9. Simultaneously, water and ion **permeability** were analyzed by measuring the corresponding fluxes across the lipid membrane. To this aim, a transition event was defined as the translocation of a given particle (a water molecule or an ion) from one compartment of the simulation box to the other across the membrane. Water transitions were counted by monitoring the position of the oxygen atom over time. No distinction was made between upward or downward transitions, the total flux being the sum of the two. To avoid miscounting that result from particle diffusion through the periodic boundary along the Z direction, a layer of 5 Å was added at the top and the bottom of the simulation box. The same setup was used to monitor the ion flux.

The configuration of the ligand-binding sites (i.e. the orthosteric and the allosteric transmembrane sites) were analyzed by monitoring one or more characteristic **distances** between residues selected based on their interaction with L-glutamate and ivermectin in the X-ray structure of the active state [7]. For the orthosteric site, the distance between the C_*α*_ atoms of Ser 150 (+) and Arg 56 (-) was used. For the allosteric site, following the work in Ref. [26] the distance between the C_*α*_ atoms of Gly 281 (+) and Leu 218 (-) was monitored. (+) and (-) refer to the principal and the complementary subunit, respectively.

In the L-Glu* simulations, the position of L-Glu was controlled by restraining the distance between its side-chain carboxylic oxygens and the basic nitrogens of Arg56 (-) and its aminic nitrogen with the geometrical center of the aromatic ring of residues Tyr 200 and Tyr 151 to the crystallographic binding mode (PDB 3RIF, see S4 Fig). To this aim, harmonic restraints with a force constant of 10 kcal/mol/Å^2^ were introduced [47].

### Software and codes used

Most of the trajectory handling and data extraction were done using Wordom [48, 49], VMD 1.9 and the python scientific packages Scipy, Numpy [50] and MDTraj [51]. All the plots were done using the python library Matplotlib 2.0 [52].

## Supporting information

S1 Table Summary of the MD simulations presented in the *Main Text*. The length of the various runs is given in nanoseconds.
(TIF)

S2 Table Characteristic distances to monitor the configuration of the orthosteric and the allosteric transmembrane sites. For the orthosteric site, the distance between the C_*α*_ atoms of Ser 150 (+) and Arg 56 (-) was used. For the allosteric site, the distance between the C_*α*_ atoms of Gly 281 (+) and Leu 218 (-) was monitored. Average values and standard deviations are given for all MD simulations presented in the *Main Text*.
(TIF)

S3 Table Average values and standard deviations for all observables discussed in *Main Text*. (TIF)

S4 Table Residues in GluCl apo (PDB 4TNV) predicted to deviate from their standard protonation state. (TIF)

S5 Table List of flipped residues suggested by MOLPROBITY for GluCl apo (PDB 4NTV). (TIF)

S1 Fig Fig 2 of the main text showing the full time length of simulations w/o IVM runs A and B. (TIF)

S2 Fig The C_*α*_-RMSD of the EC domain from the X-ray structure of GluCL apo is shown as a function of the time for 4 simulations. (TIF)

S3 Fig Structural rearrangement of the EC/TM domains interface during ion-channel deactivation. The position of the center of mass of P268 projected on the plane of the membrane is monitored over time in the simulations of GluCl starting from the active state with (red) and without IVM run B (blue) and from the resting state (green).
(TIF)

S4 Fig Distances restrained in the L-Glu* simulation. Reference values were taken from the crystal structure (3RIF). Identical distances were restrained for the 5 ligands.
(TIF)

S5 Fig M2-M3 coupling during pore closing. The separation between transmembrane helices M2 and M3 along the three simulations of GluCl is shown as a function of time; the active state with (red) and without (blue) IVM and the resting state (green). Results are shown only for simulation A, run B leading to similar conclusions. The dotted lines correspond to the M2-M3 distance measured in the crystal structures of the locally closed (LC) state of GLIC (PDB 4NPP), the resting state of GLIC (PDB 4NPQ) and the closed-channel state of ELIC (PDB 2VL0).
(TIF)

S6 Fig Orthosteric site topology. The distribution of the distance between the C_*α*_ atoms of the residues Ser 150 (+) and Arg 56 (-) averaged over the five subunits is shown for the simulations of GluCl apo (green), GluCl active with IVM bound (red) and IVM removed (dark and light blue), and GluCl active with harmonic restraints on L-Glu (orange).
(TIF)

S7 Fig Evolution of the distance between the center of mass of each strictly conserved proline residue (P268 in GluCl) and the center of the pore. Results per subunit are given in colors, the average profile is in black. Data are shown only for run A, run B leading to the same conclusions.
(TIF)

S8 Fig Tilt and twist correlation. The azimuthal component of the tilt is plotted against the twist. In green and yellow diamonds are shown respectively the crystal structure of GluCl apo (PDB 4TNV) and GluCl bound to IVM (PDB 3RIF).
(TIF)

S9 Fig Correlation between the opening of the pore at the constriction point and the average distance of P268 to the center of the pore. Are shown in blue the results before the full twisting of the receptor (0–400ns) and in cyan the results after the twisting (400–2500ns). Results are shown only for run A, run B leading to the same conclusions.
(TIF)

S10 Fig Autocorrelation function (acf) of the RMSD for the two reference simulations, i.e., GluCl apo and GluCl with IVM and L-Glu bound. The exponential fits are shown in dotted lines. Due to the way the acf was computed only half of the simulations are shown. One clearly sees that it takes a significantly longer time for the EC domain to converge that for the TM domain. Also, this data show that the systems reach an equilibrium at about 100 ns for IVM bound and about 40 ns for the TM GluCl with IVM bound. Finally it is interesting to mention that the EC domain of GluCl apo seems to not be yet at equilibrium, this can be explained by the absence of ligand at the level of the orthosteric site making the sampling of all possible configurations more difficult.
(TIF)

S11 Fig C_*α*_-RMSD along the MD trajectories of the two reference simulations, i.e., GluCl apo and GluCl active with IVM and L-Glu bound. Average values are represented by dotted lines.
(TIF)

S12 Fig Closing of the pore. When IVM is removed the pore gradually closes at 9′ in run B as well. The plots are average over the period of time given in the caption. To facilitate the understanding of the reader only the TM domain is shown.
(TIF)

S13 Fig Time series of the C_*α*_-RMSD of the EC (top) and TM (bottom) domains from the X-ray structure of GluCl active with IVM bound (PDBID 3RIF) for the four simulations presented in the *Main Text*. (TIF)

S14 Fig Orientation of the Leu254 side chain forming the constriction point. *Cis* and *trans* configuration ranges are displayed respectively by pink and yellow colors. One clearly sees that all 4 systems mainly populated a *trans* configuration of the *χ* angle, which corresponds to the side chain oriented toward the lumen of the pore. Interestingly, GluCl active (with the wider constriction point diameter) shows a small population of *χ* angle is the *cis* configuration. The *χ* angle is defined as the dihedral angle between the following four atoms: C_*α*_, C_*β*_, C_*γ*_, and the nitrogen involved in the peptide bond.
(TIF)

S15 Fig β-sheet expansion. This distance is measured between the C_*α*_ of the residues R211 and V44 in GluCl, which correspond to R192 and D32 in GLIC. No significant expansion is seen upon closing unlike described in Ref. [31].
(TIF)

S16 Fig Representation of the water distribution along the pore as function of time for four different systems. The zero of the Y axis is centered on the 9′ residues. Active (with IVM), the first 400 ns of w/o IVM run A, and the first 1*μs* of w/o IVM run B simulations show a full wetting of the pore. GluCl apo, and the second part of both simulations in which IVM was removed show a partial dewetting of the ion pore corresponding to a dehydrated stretch of ∼ 5 Å; see S17 Fig.
(TIF)

S17 Fig Comparison between the HOLE profile and the normalized water density distribution inside the pore for the four systems in S16 Fig. The zero of the *Z coord* corresponds to the position of residues at 9′. The dashed black line represents the radius of a water molecule (1.5 Å). The dehydration stretch is measured as the extension of the pore region where the water density is below one hundredth of the bulk. The length of the dehydrated stretch is 8.4, 4.8 and 4.6 Å in apo, w/o IVM run A and run B, respectively.
(TIF)

S1 Video Visualization of the intersubunit variability for two simulations of GluCl. In shades of red is displayed GluCl bound to IVM and in shades of blue GluCl w/o IVM. One can see from the top view the significant difference in motion of the two systems. The former showing no blooming and coupled motions of the subunits and the latter showing decoupled motions of the subunits, thus different blooming angles among the 5 subunits. (File size is 18.4 Mb).
(MP4)

S2 Video Visualization of the pore-closing transition illustrating/emphasizing the global *twisting* of the receptor followed by a radial expansion or *blooming* of the EC domain. The conformation of the active state corresponding to the first frame of the simulation is shown in grey. (File size is 18.5 Mb).
(MP4)
