# Supplementary figures and images for: Un-gating and allosteric modulation of a pentameric ligand-gated ion channel captured by molecular dynamics
Source: PLoS Comput Biol. 2017 Oct 25;13(10):e1005784. doi: 10.1371/journal.pcbi.1005784 (PMC5673239; doi:10.1371/journal.pcbi.1005784)

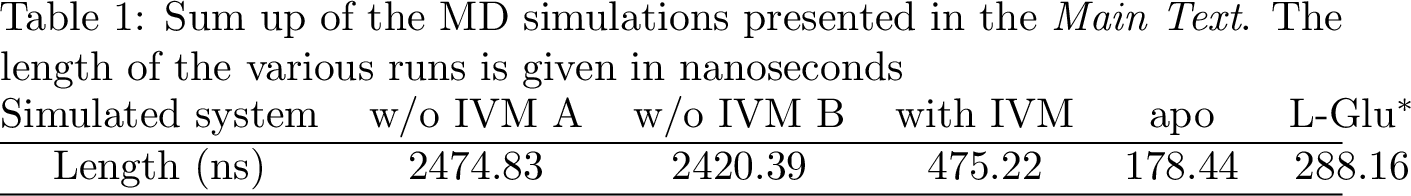

Supplement: S1 Table — The length of the various runs is given in nanoseconds. (TIF) [file pcbi.1005784.s001.tif]

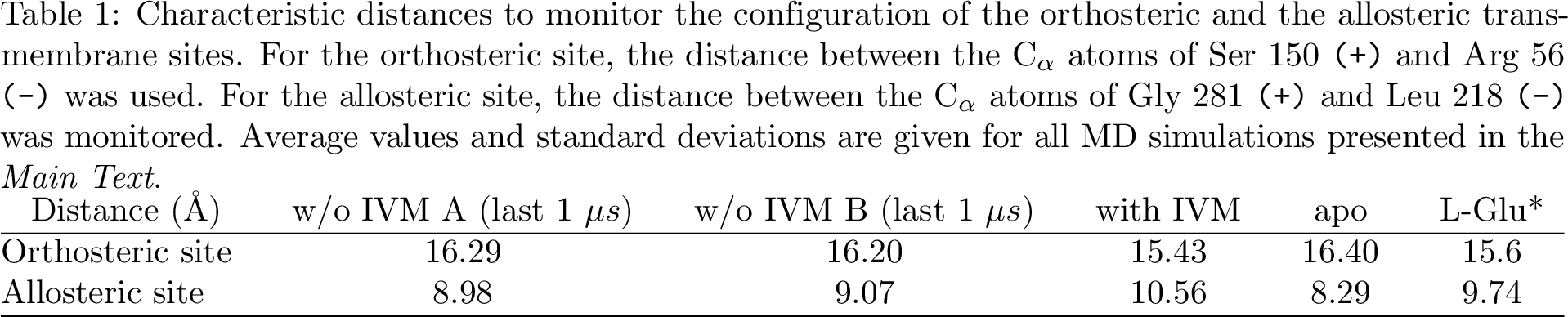

Supplement: S2 Table — For the orthosteric site, the distance between the Cα atoms of Ser 150 (+) and Arg 56 (-) was used. For the allosteric site, the distance between the Cα atoms of Gly 281 (+) and Leu 218 (-) was monitored. Average values and standard deviations are given for all MD simulations presented in the Main Text. (TIF) [file pcbi.1005784.s002.tif]

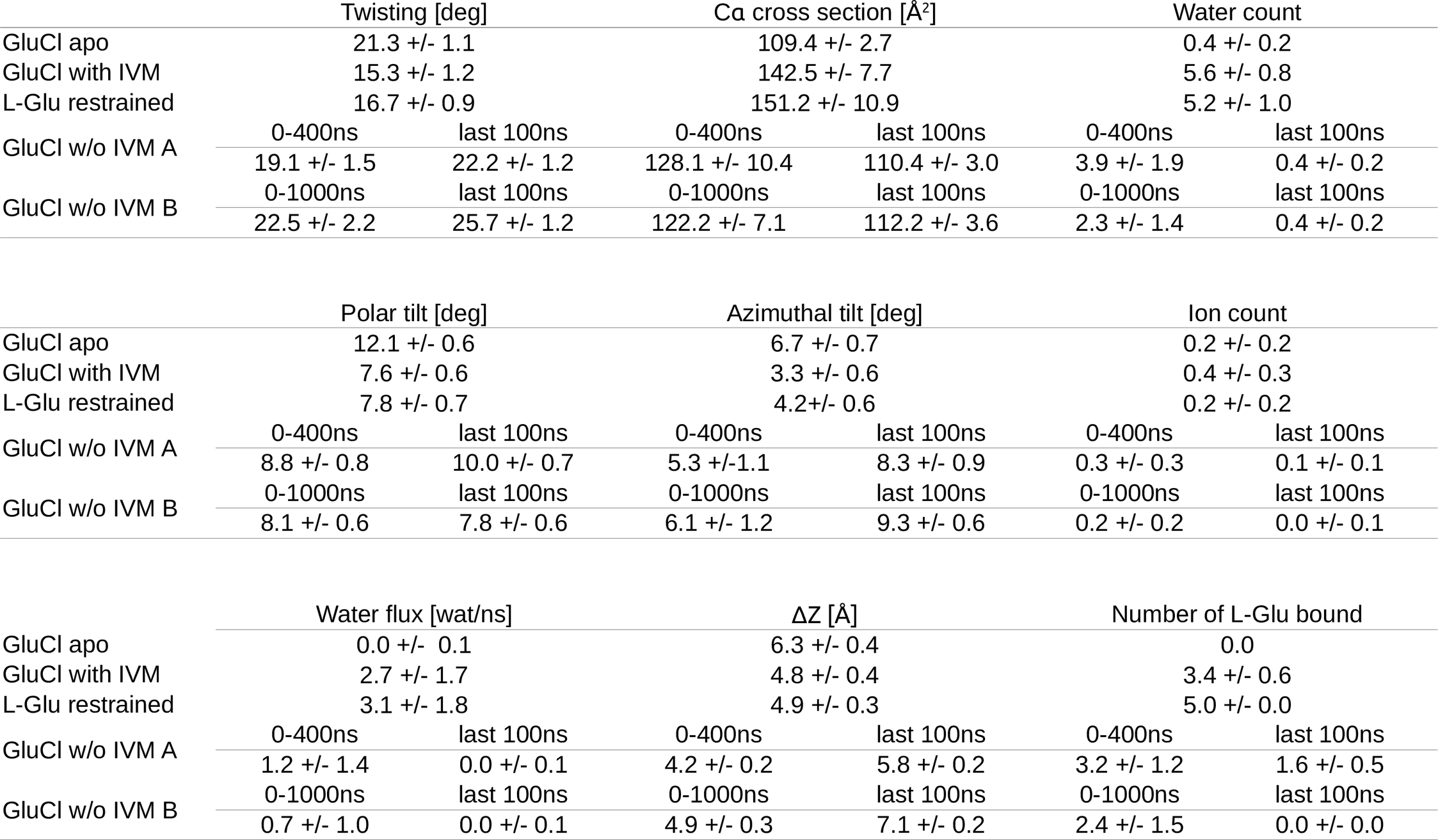

Supplement: S3 Table — (TIF) [file pcbi.1005784.s003.tif]

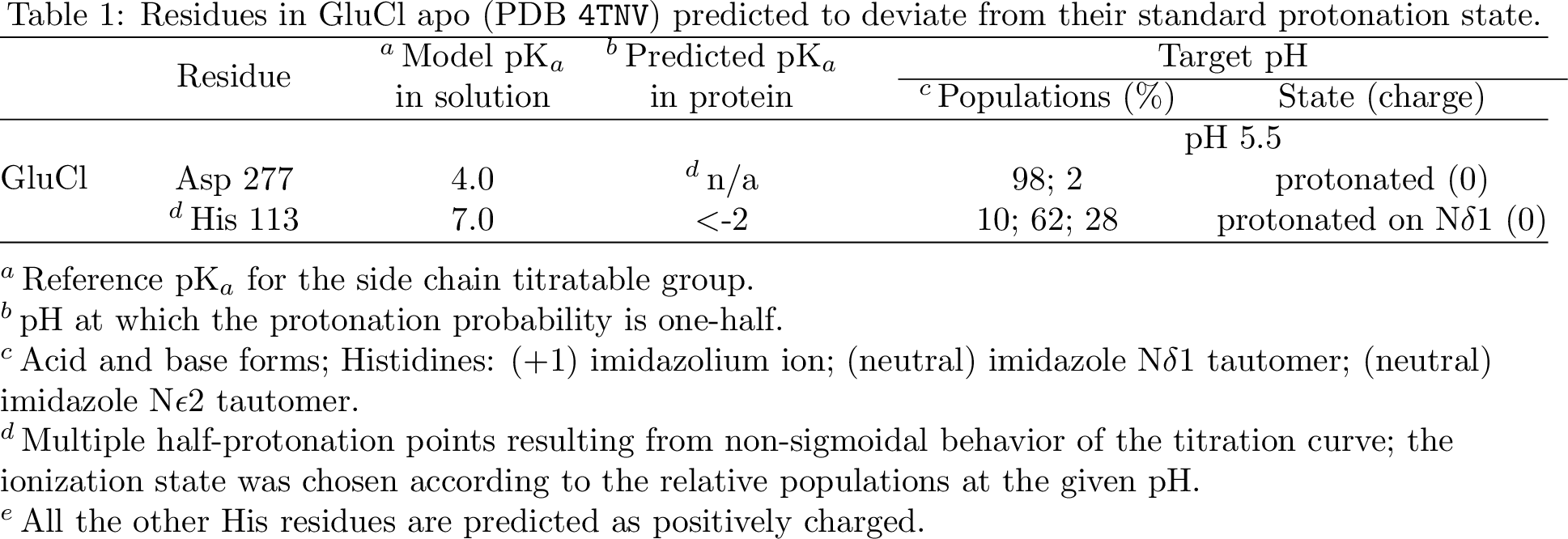

Supplement: S4 Table — (TIF) [file pcbi.1005784.s004.tif]

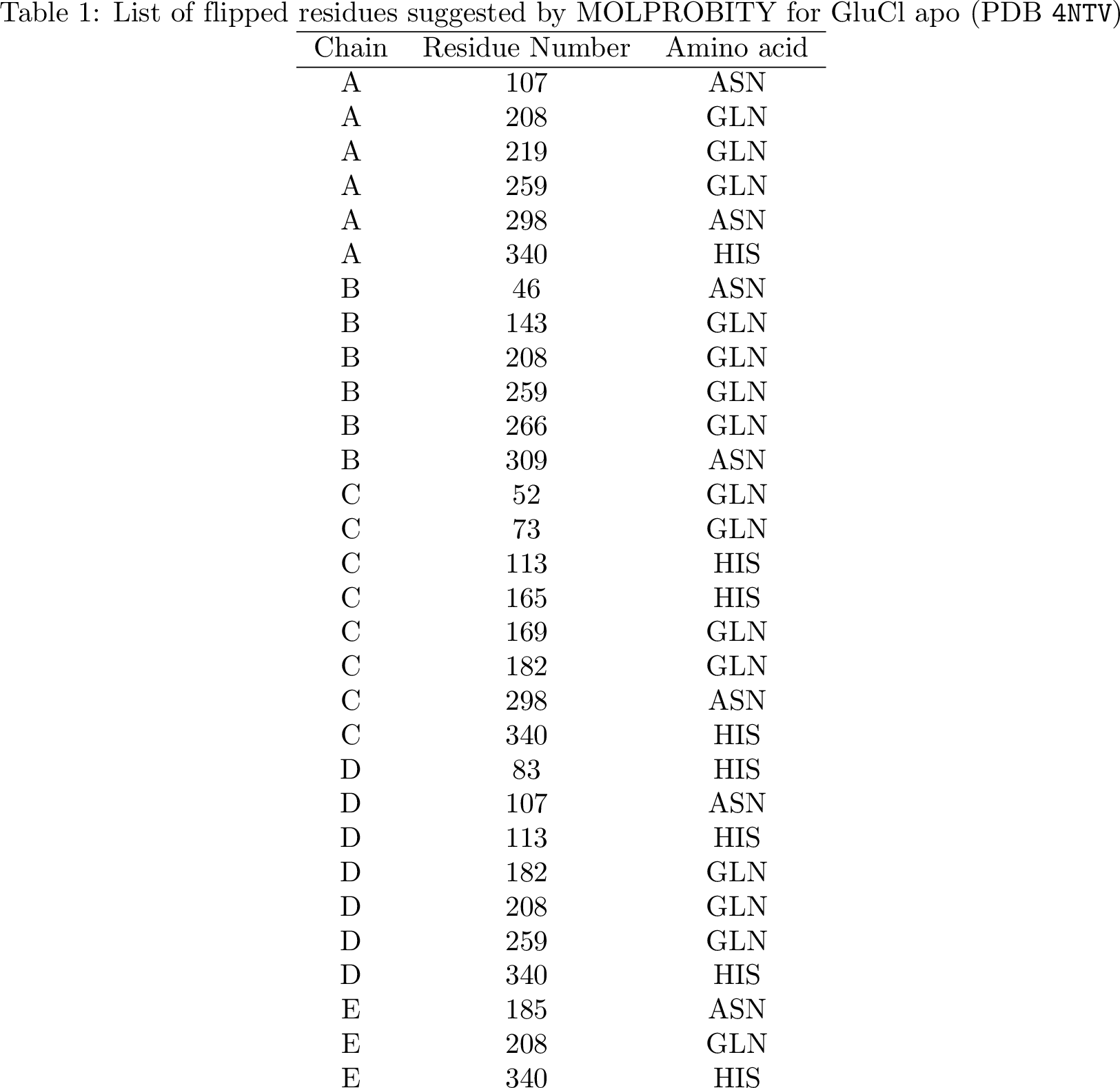

Supplement: S5 Table — (TIF) [file pcbi.1005784.s005.tif]

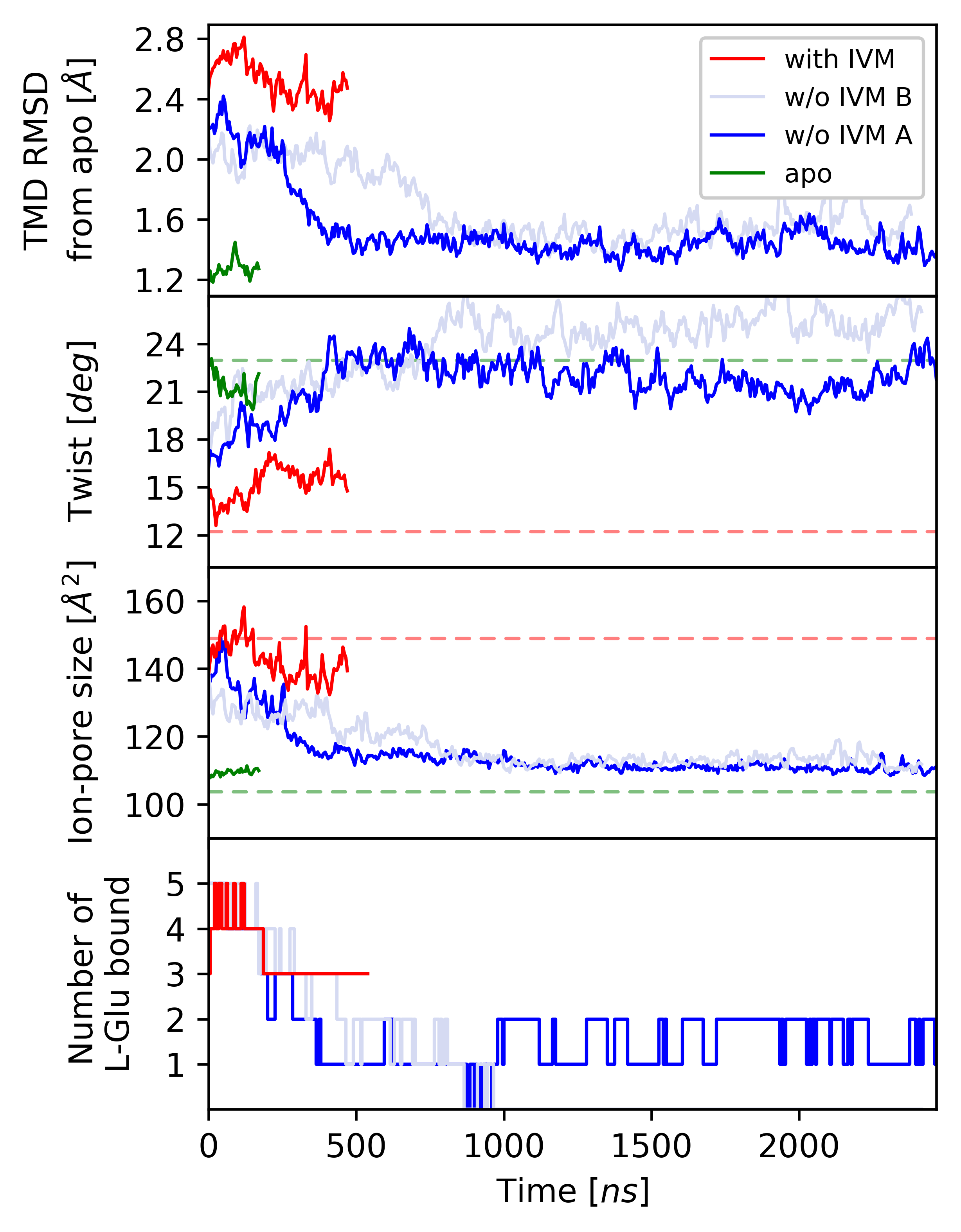

Supplement: S1 Fig — (TIF) [file pcbi.1005784.s006.tif]

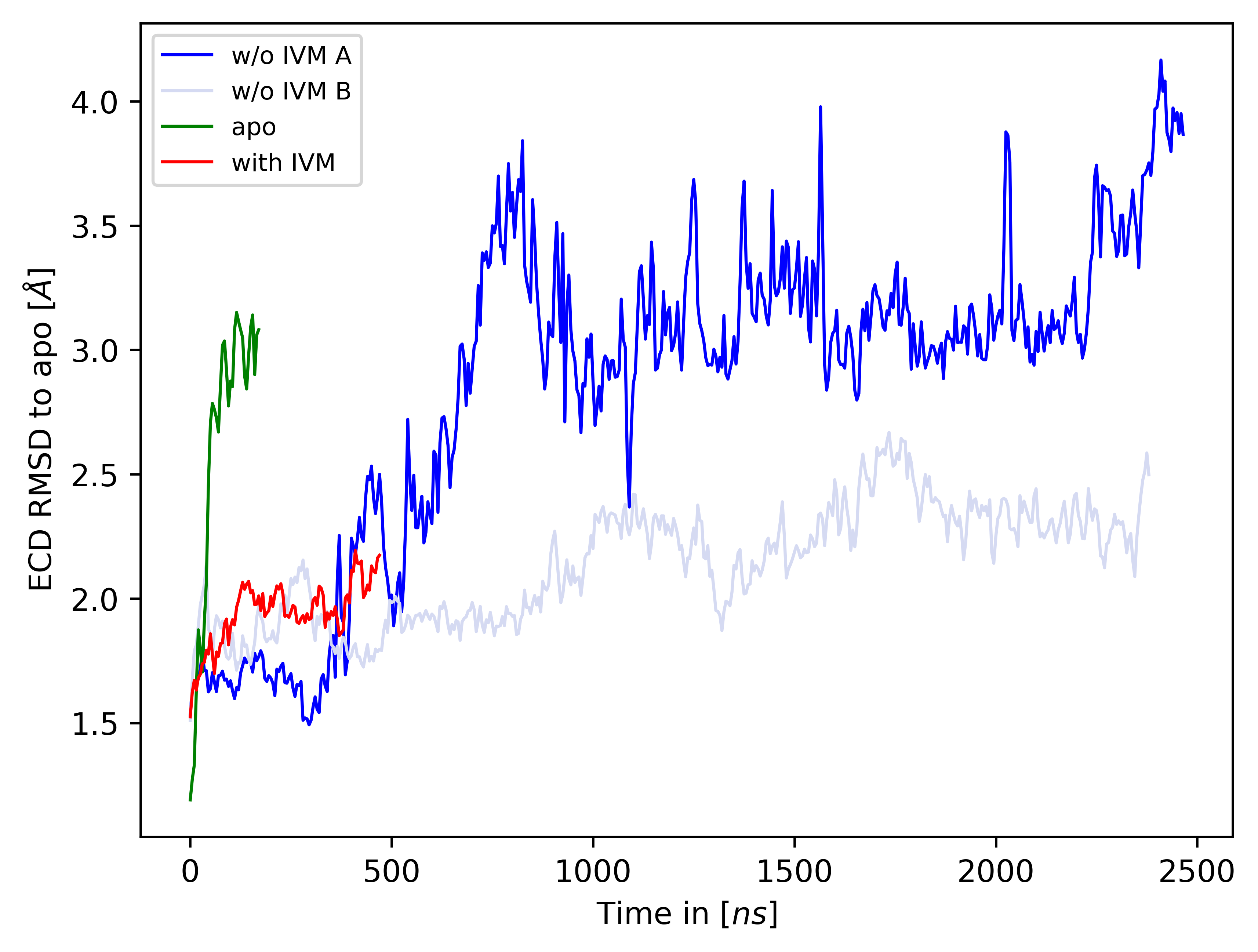

Supplement: S2 Fig — (TIF) [file pcbi.1005784.s007.tif]

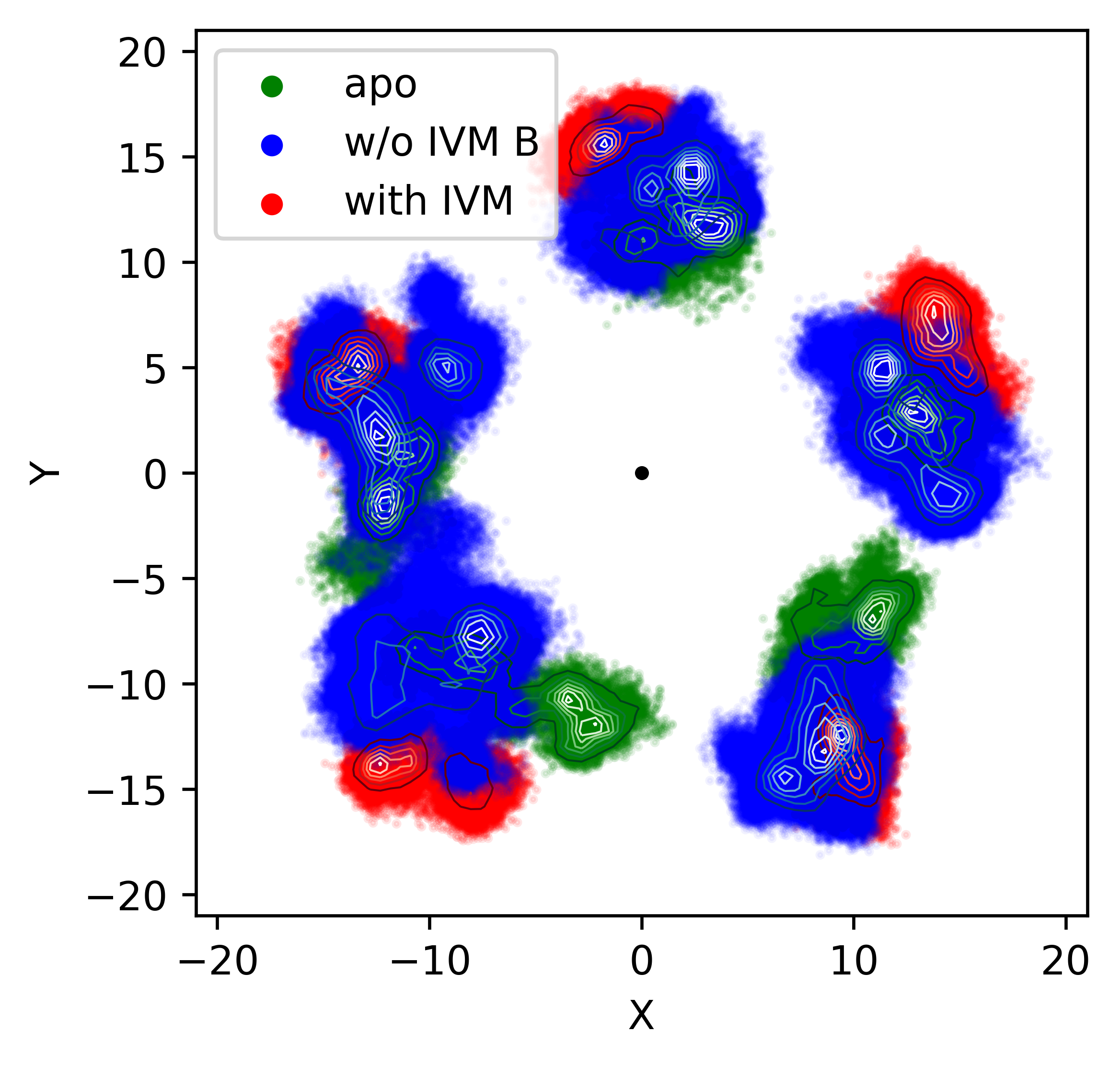

Supplement: S3 Fig — The position of the center of mass of P268 projected on the plane of the membrane is monitored over time in the simulations of GluCl starting from the active state with (red) and without IVM run B (blue) and from the resting state (green). (TIF) [file pcbi.1005784.s008.tif]

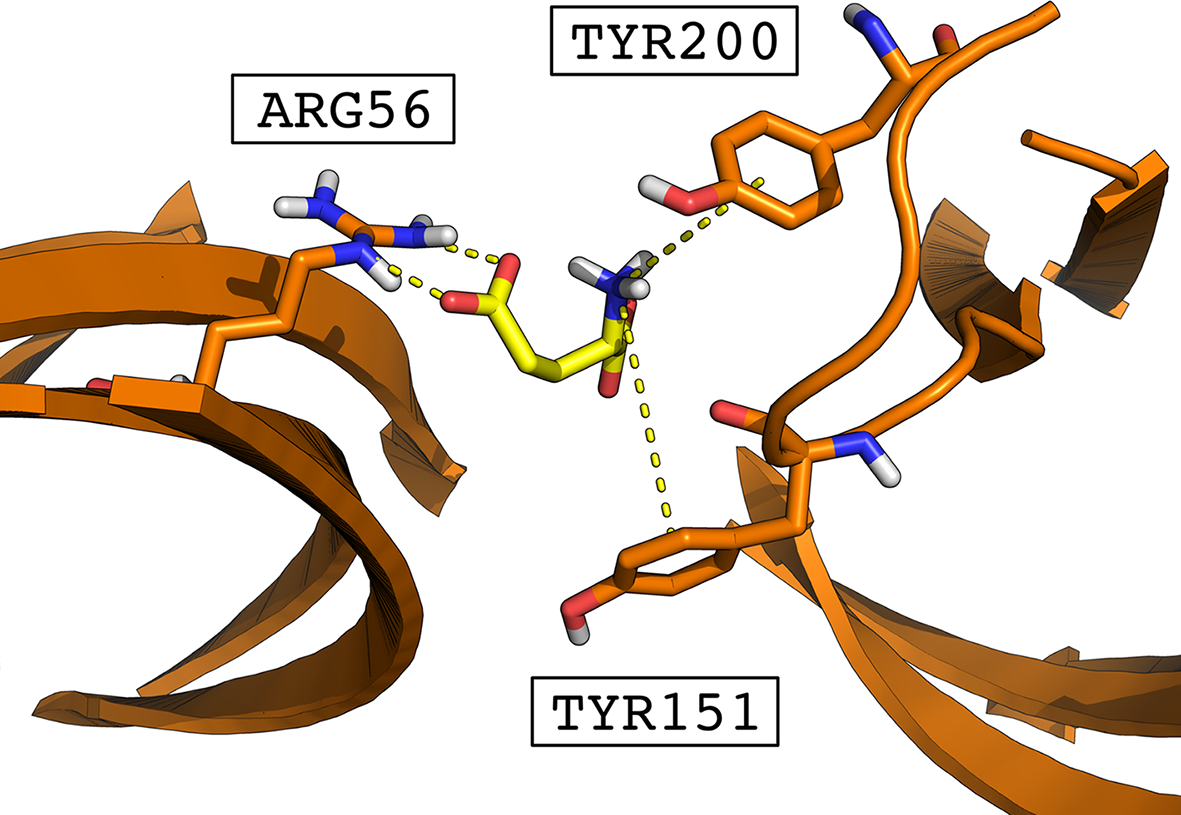

Supplement: S4 Fig — Reference values were taken from the crystal structure (3RIF). Identical distances were restrained for the 5 ligands. (TIF) [file pcbi.1005784.s009.tif]

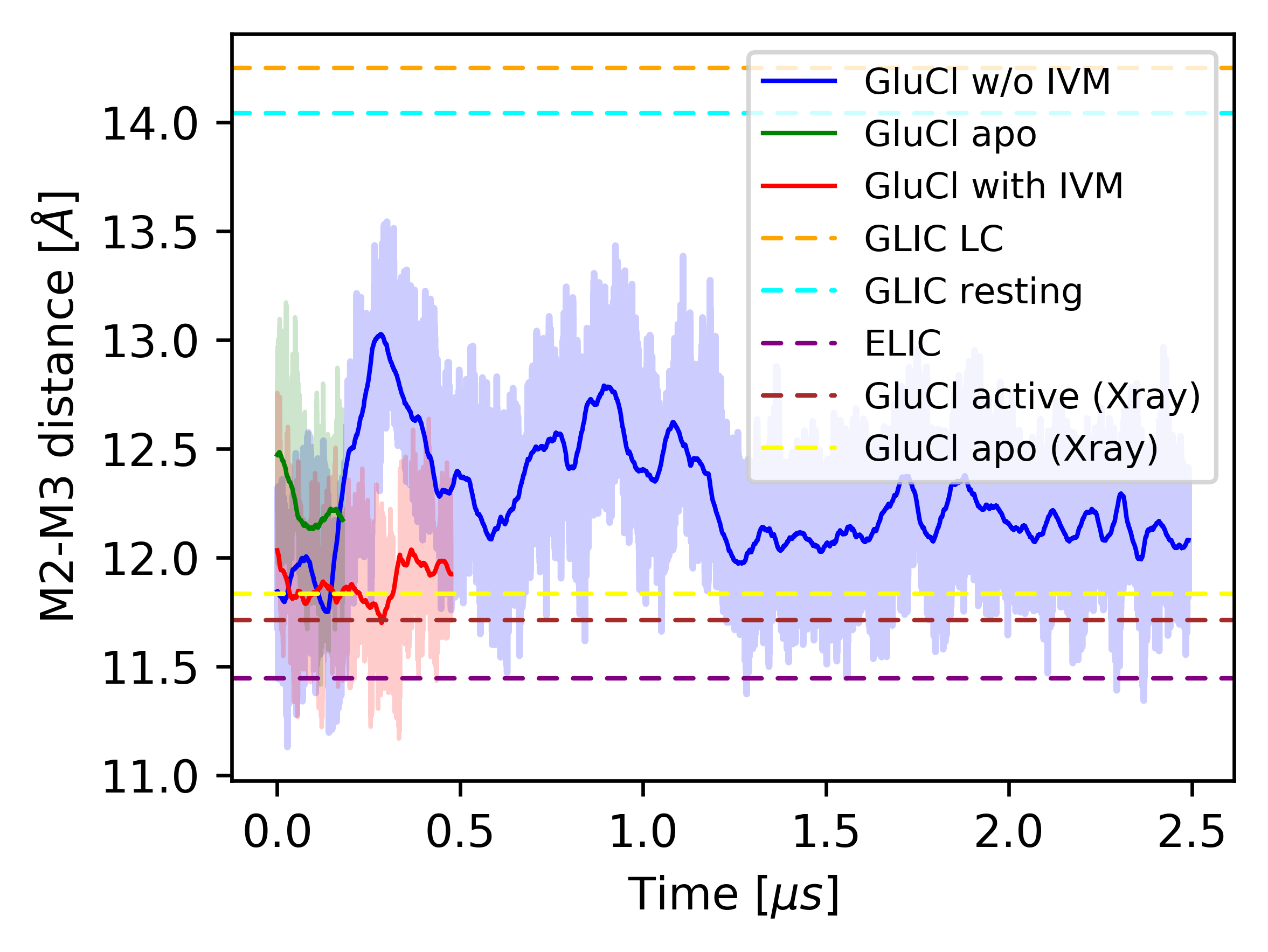

Supplement: S5 Fig — The separation between transmembrane helices M2 and M3 along the three simulations of GluCl is shown as a function of time; the active state with (red) and without (blue) IVM and the resting state (green). Results are shown only for simulation A, run B leading to similar conclusions. The dotted lines correspond to the M2-M3 distance measured in the crystal structures of the locally closed (LC) state of GLIC (PDB 4NPP), the resting state of GLIC (PDB 4NPQ) and the closed-channel state of ELIC (PDB 2VL0). (TIF) [file pcbi.1005784.s010.tif]

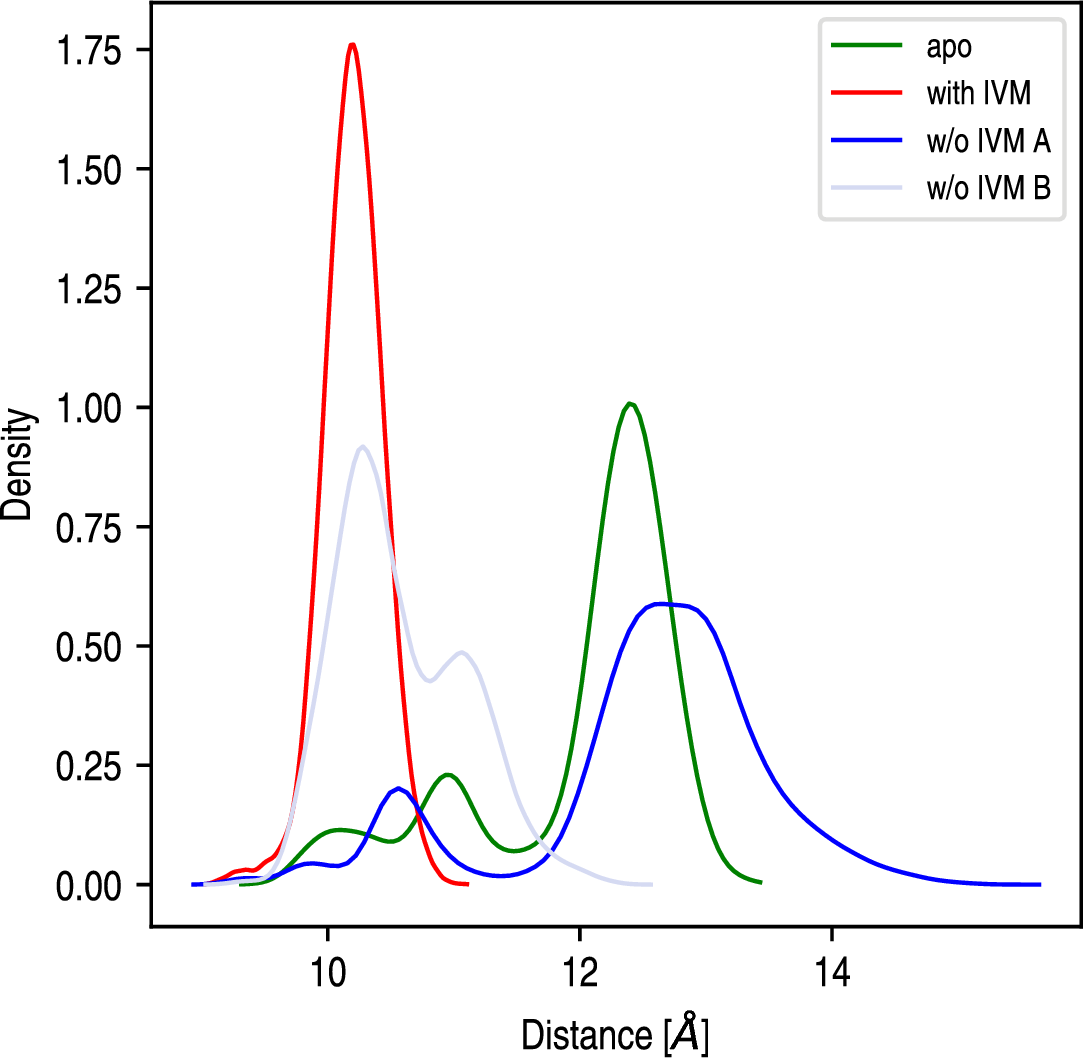

Supplement: S6 Fig — The distribution of the distance between the Cα atoms of the residues Ser 150 (+) and Arg 56 (-) averaged over the five subunits is shown for the simulations of GluCl apo (green), GluCl active with IVM bound (red) and IVM removed (dark and light blue), and GluCl active with harmonic restraints on L-Glu (orange). (TIF) [file pcbi.1005784.s011.tif]

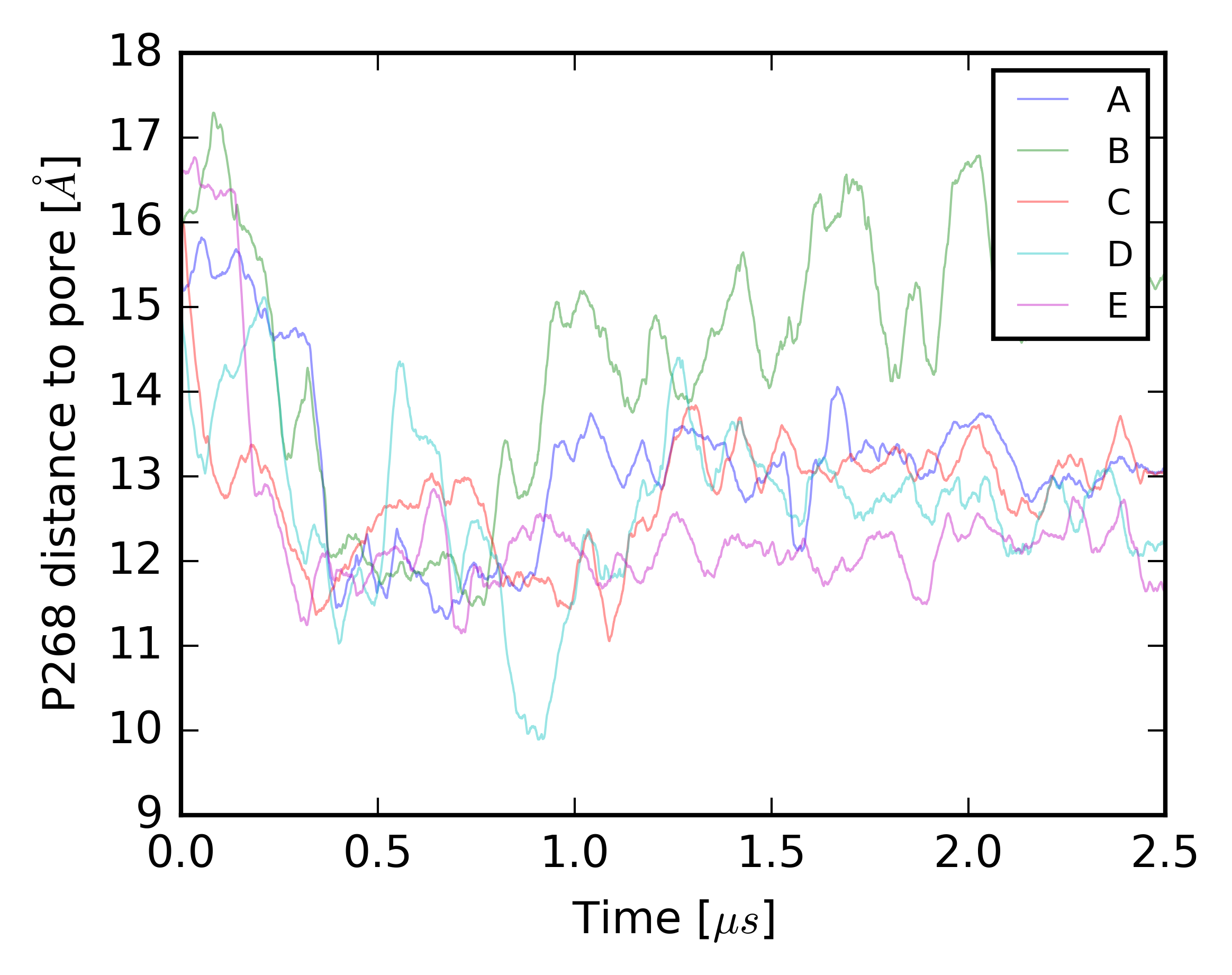

Supplement: S7 Fig — Results per subunit are given in colors, the average profile is in black. Data are shown only for run A, run B leading to the same conclusions. (TIF) [file pcbi.1005784.s012.tif]

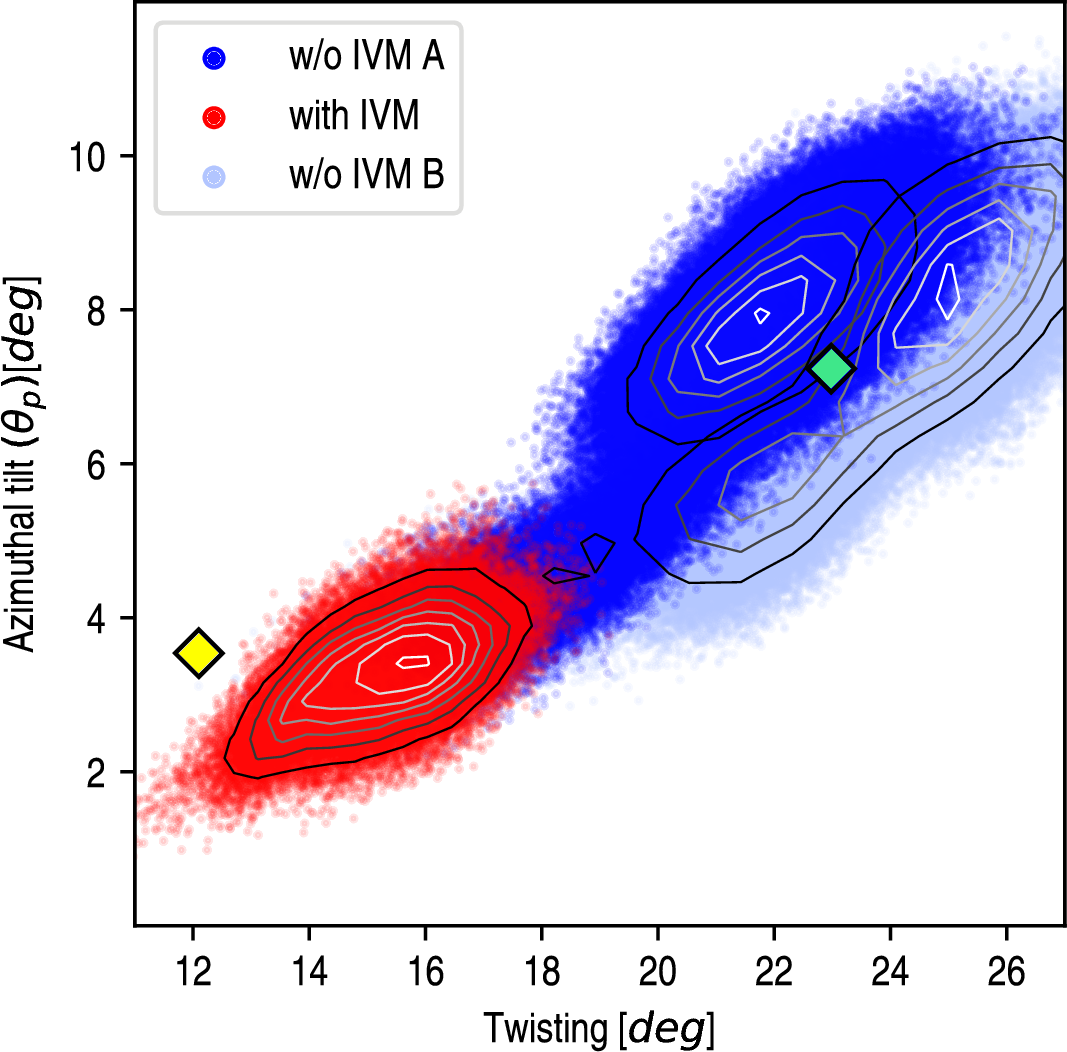

Supplement: S8 Fig — The azimuthal component of the tilt is plotted against the twist. In green and yellow diamonds are shown respectively the crystal structure of GluCl apo (PDB 4TNV) and GluCl bound to IVM (PDB 3RIF). (TIF) [file pcbi.1005784.s013.tif]

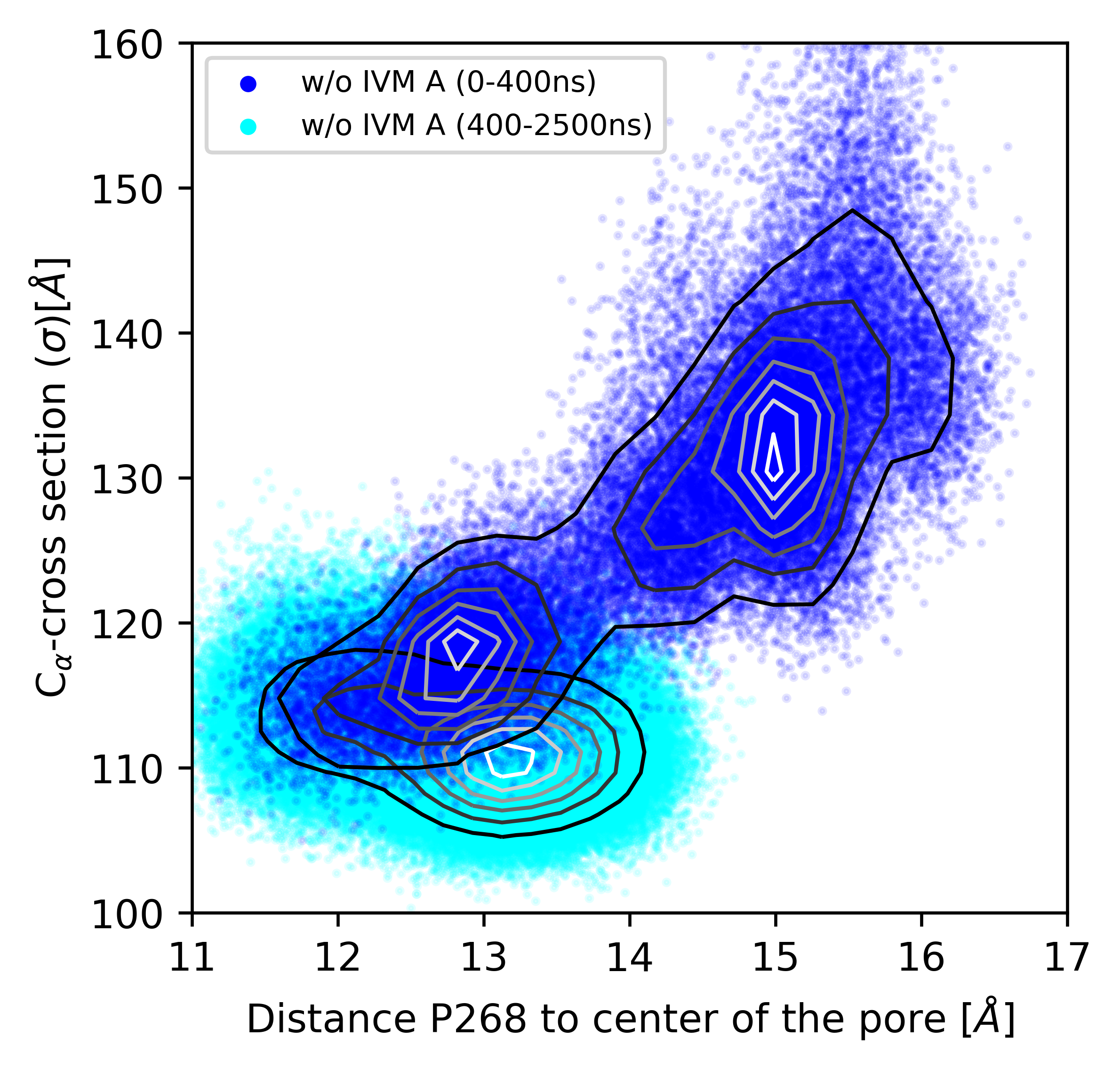

Supplement: S9 Fig — Are shown in blue the results before the full twisting of the receptor (0–400ns) and in cyan the results after the twisting (400–2500ns). Results are shown only for run A, run B leading to the same conclusions. (TIF) [file pcbi.1005784.s014.tif]

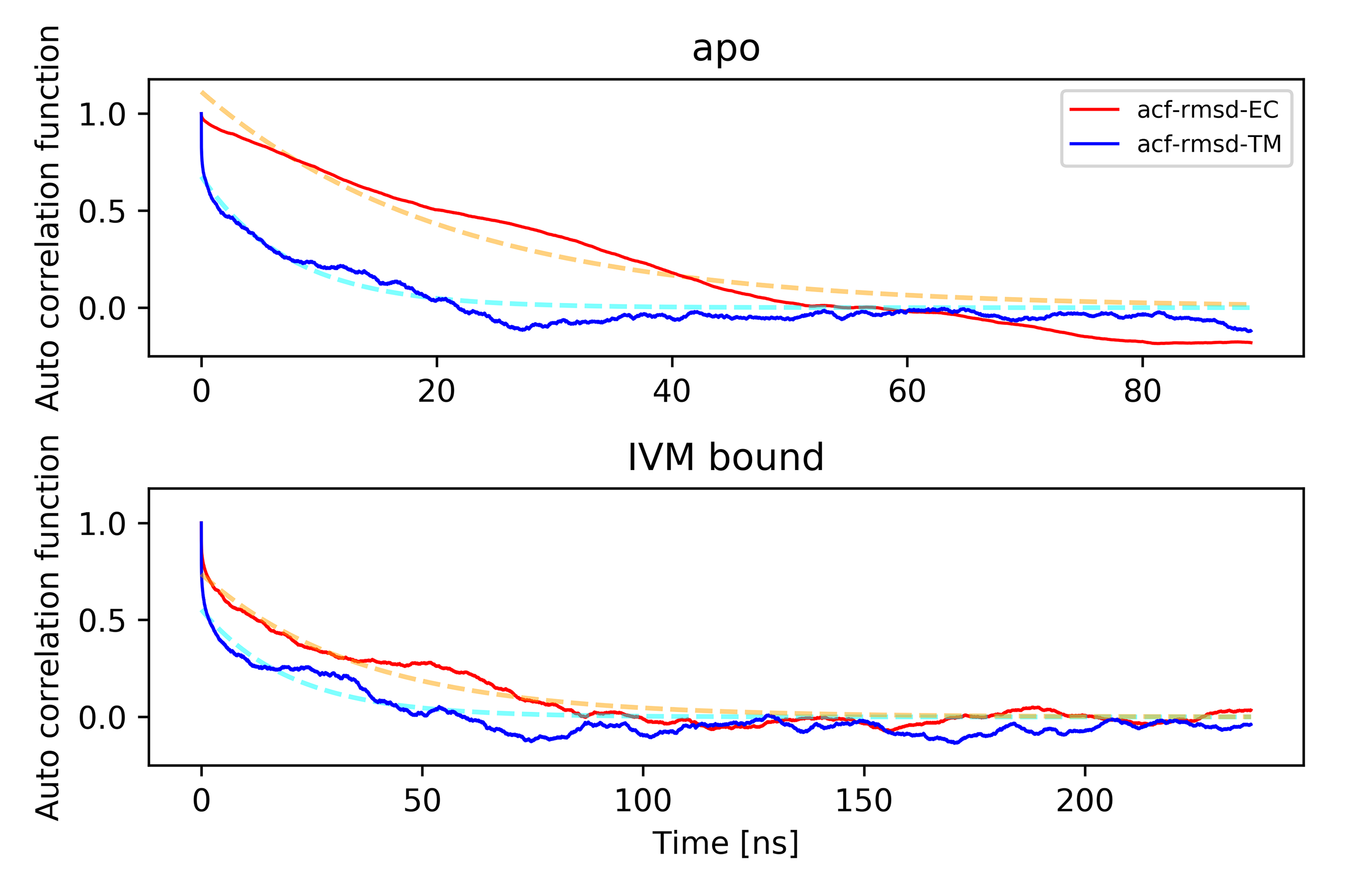

Supplement: S10 Fig — The exponential fits are shown in dotted lines. Due to the way the acf was computed only half of the simulations are shown. One clearly sees that it takes a significantly longer time for the EC domain to converge that for the TM domain. Also, this data show that the systems reach an equilibrium at about 100 ns for IVM bound and about 40 ns for the TM GluCl with IVM bound. Finally it is interesting to mention that the EC domain of GluCl apo seems to not be yet at equilibrium, this can be explained by the absence of ligand at the level of the orthosteric site making the sampling of all possible configurations more difficult. (TIF) [file pcbi.1005784.s015.tif]

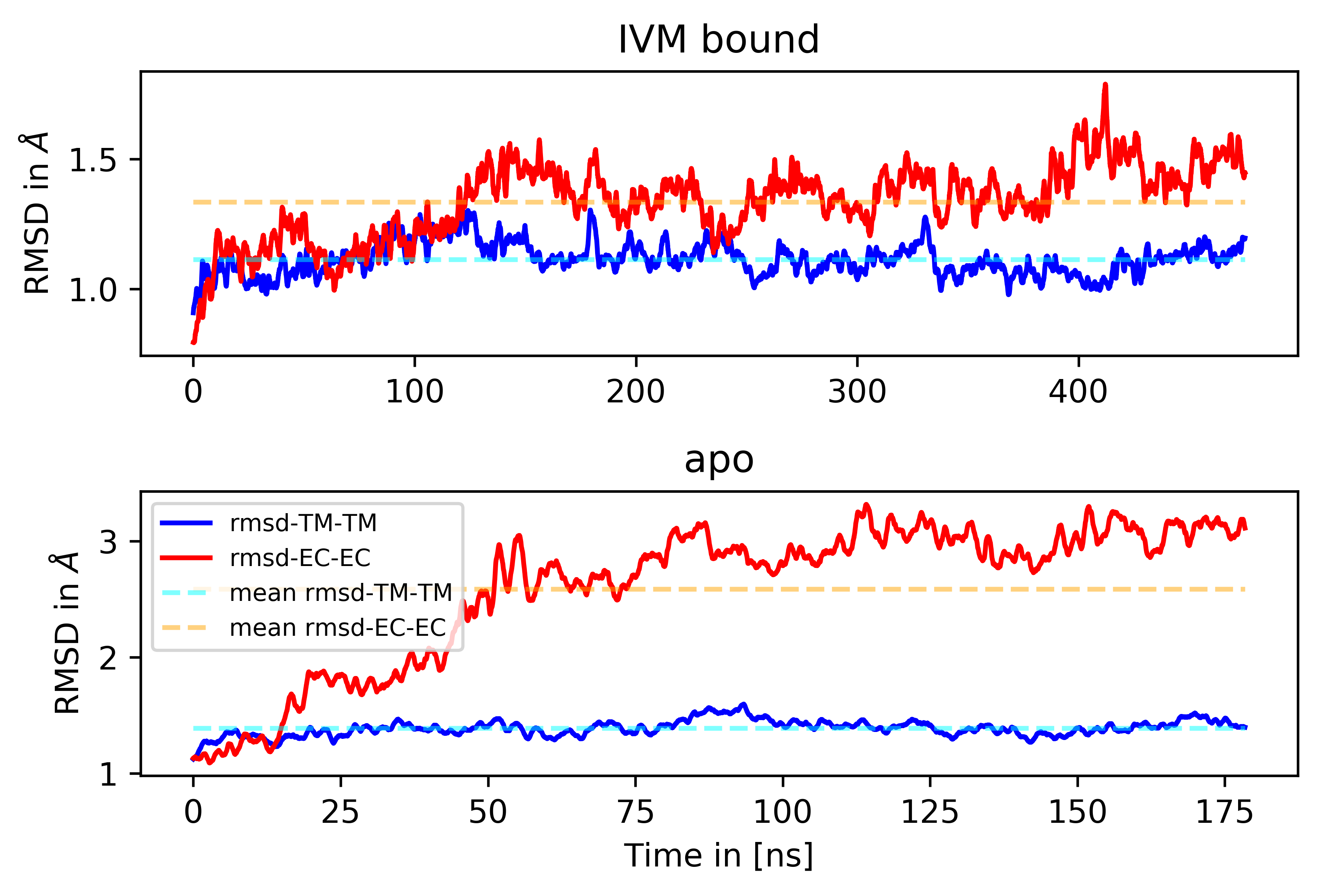

Supplement: S11 Fig — Average values are represented by dotted lines. (TIF) [file pcbi.1005784.s016.tif]

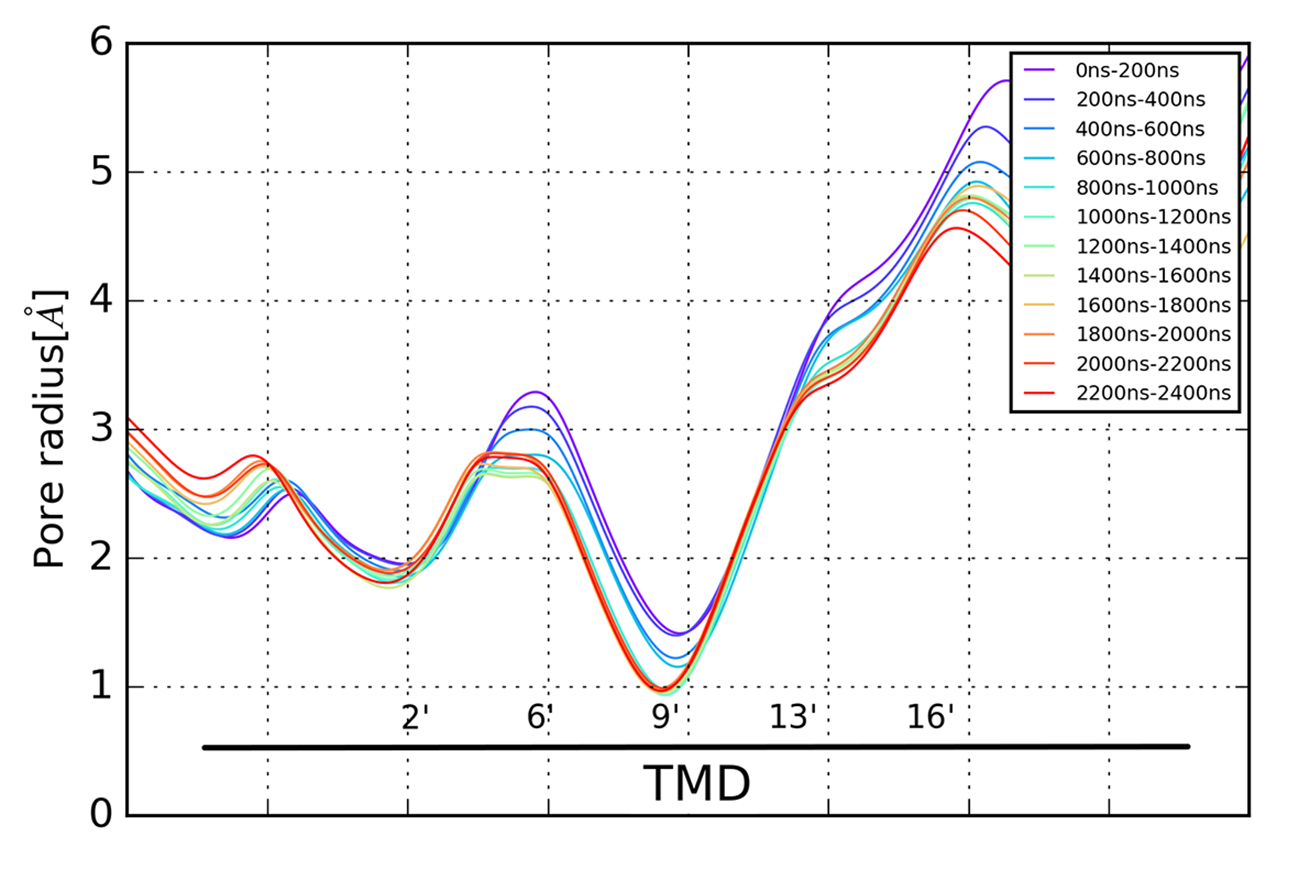

Supplement: S12 Fig — When IVM is removed the pore gradually closes at 9′ in run B as well. The plots are average over the period of time given in the caption. To facilitate the understanding of the reader only the TM domain is shown. (TIF) [file pcbi.1005784.s017.tif]

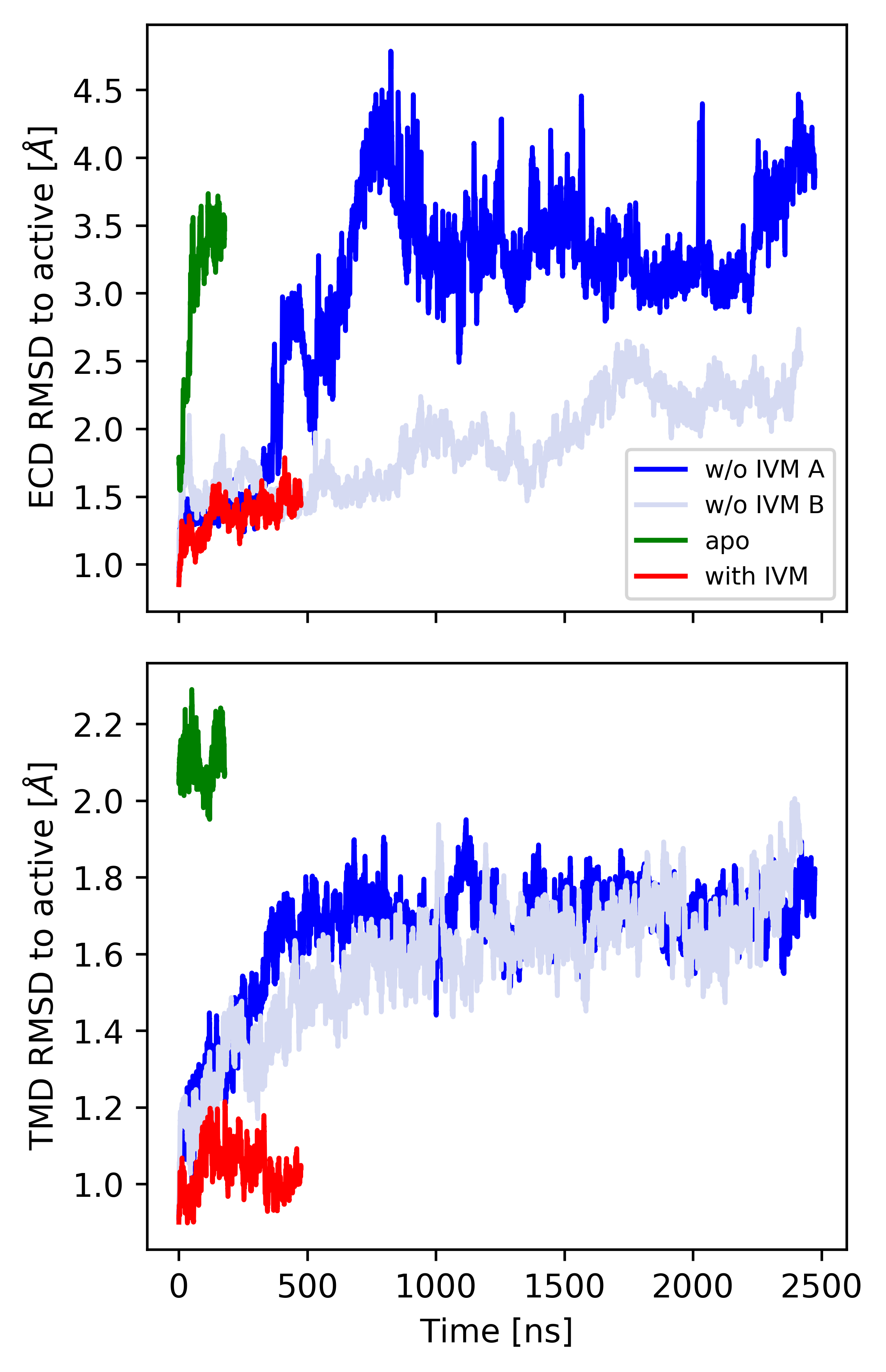

Supplement: S13 Fig — (TIF) [file pcbi.1005784.s018.tif]

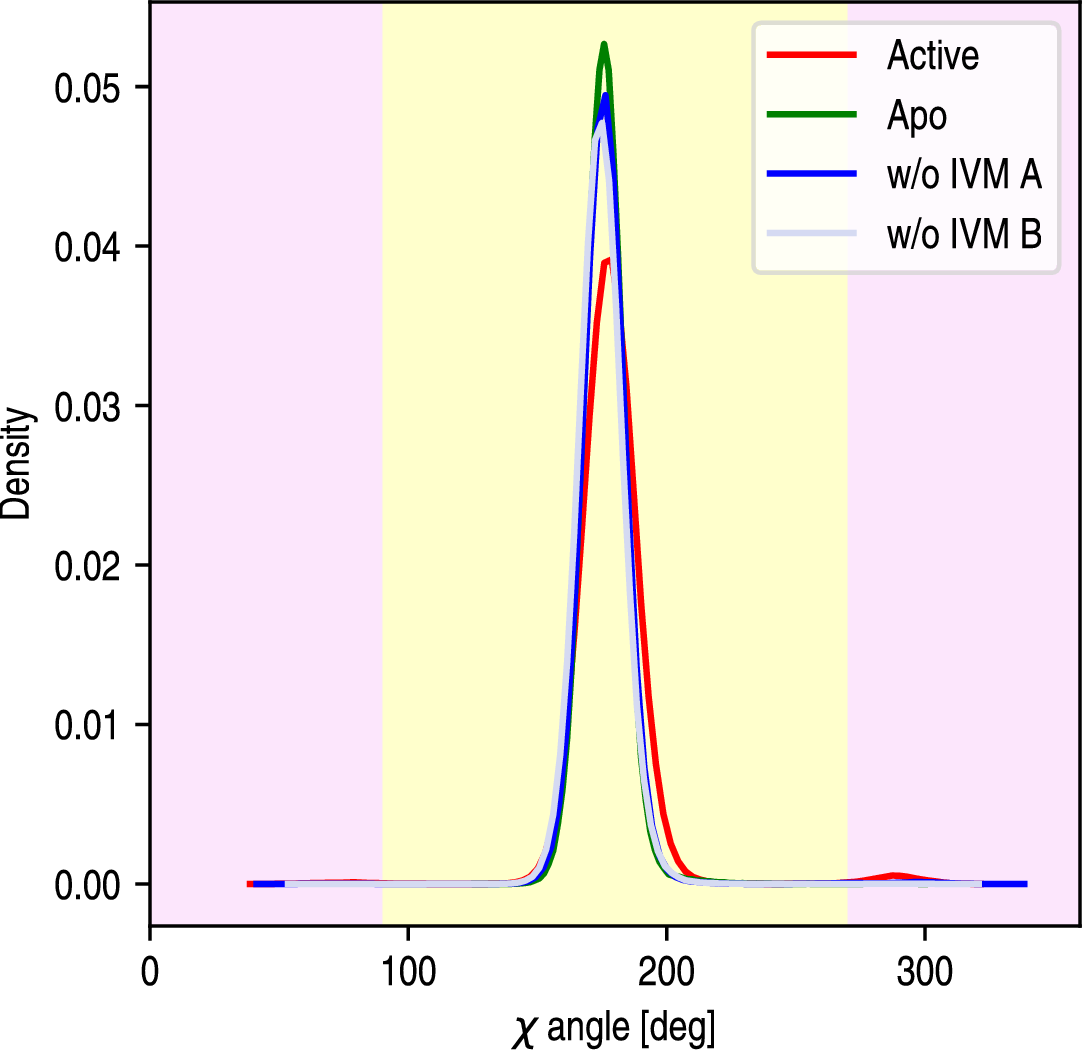

Supplement: S14 Fig — Cis and trans configuration ranges are displayed respectively by pink and yellow colors. One clearly sees that all 4 systems mainly populated a trans configuration of the χ angle, which corresponds to the side chain oriented toward the lumen of the pore. Interestingly, GluCl active (with the wider constriction point diameter) shows a small population of χ angle is the cis configuration. The χ angle is defined as the dihedral angle between the following four atoms: Cα, Cβ, Cγ, and the nitrogen involved in the peptide bond. (TIF) [file pcbi.1005784.s019.tif]

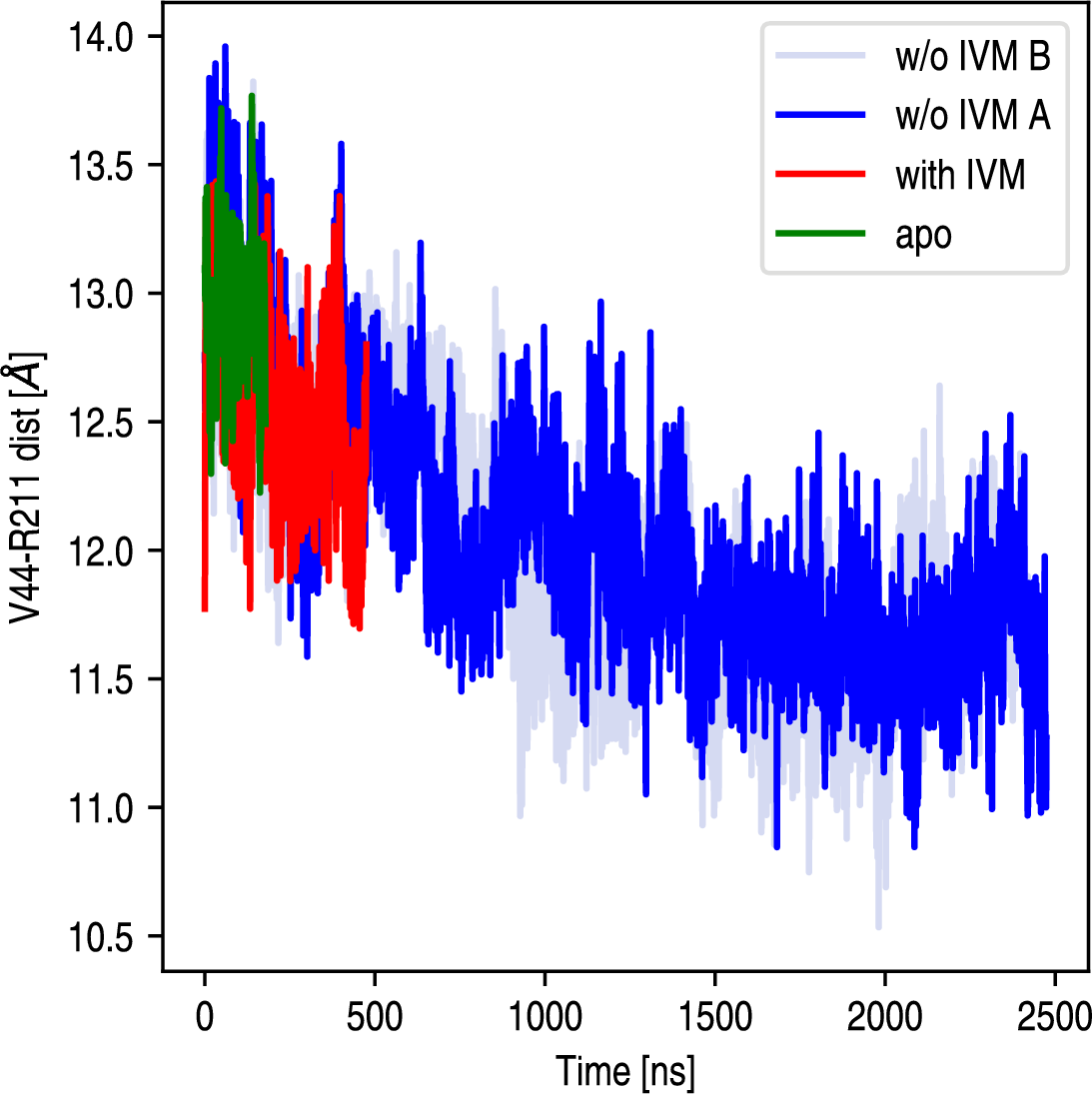

Supplement: S15 Fig — This distance is measured between the Cα of the residues R211 and V44 in GluCl, which correspond to R192 and D32 in GLIC. No significant expansion is seen upon closing unlike described in Ref. [31]. (TIF) [file pcbi.1005784.s020.tif]

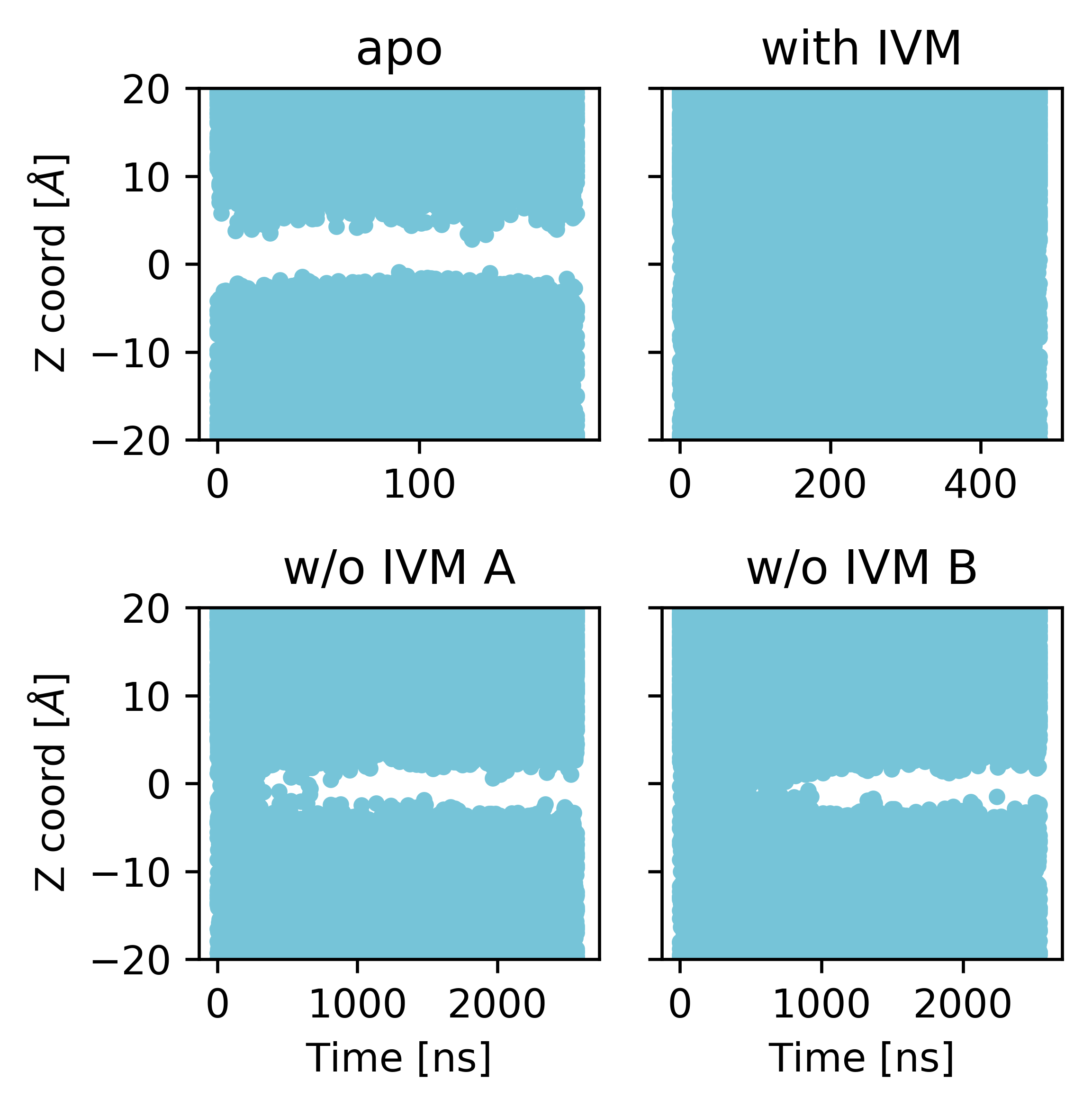

Supplement: S16 Fig — The zero of the Y axis is centered on the 9′ residues. Active (with IVM), the first 400 ns of w/o IVM run A, and the first 1μs of w/o IVM run B simulations show a full wetting of the pore. GluCl apo, and the second part of both simulations in which IVM was removed show a partial dewetting of the ion pore corresponding to a dehydrated stretch of ∼ 5 Å; see S17 Fig. (TIF) [file pcbi.1005784.s021.tif]

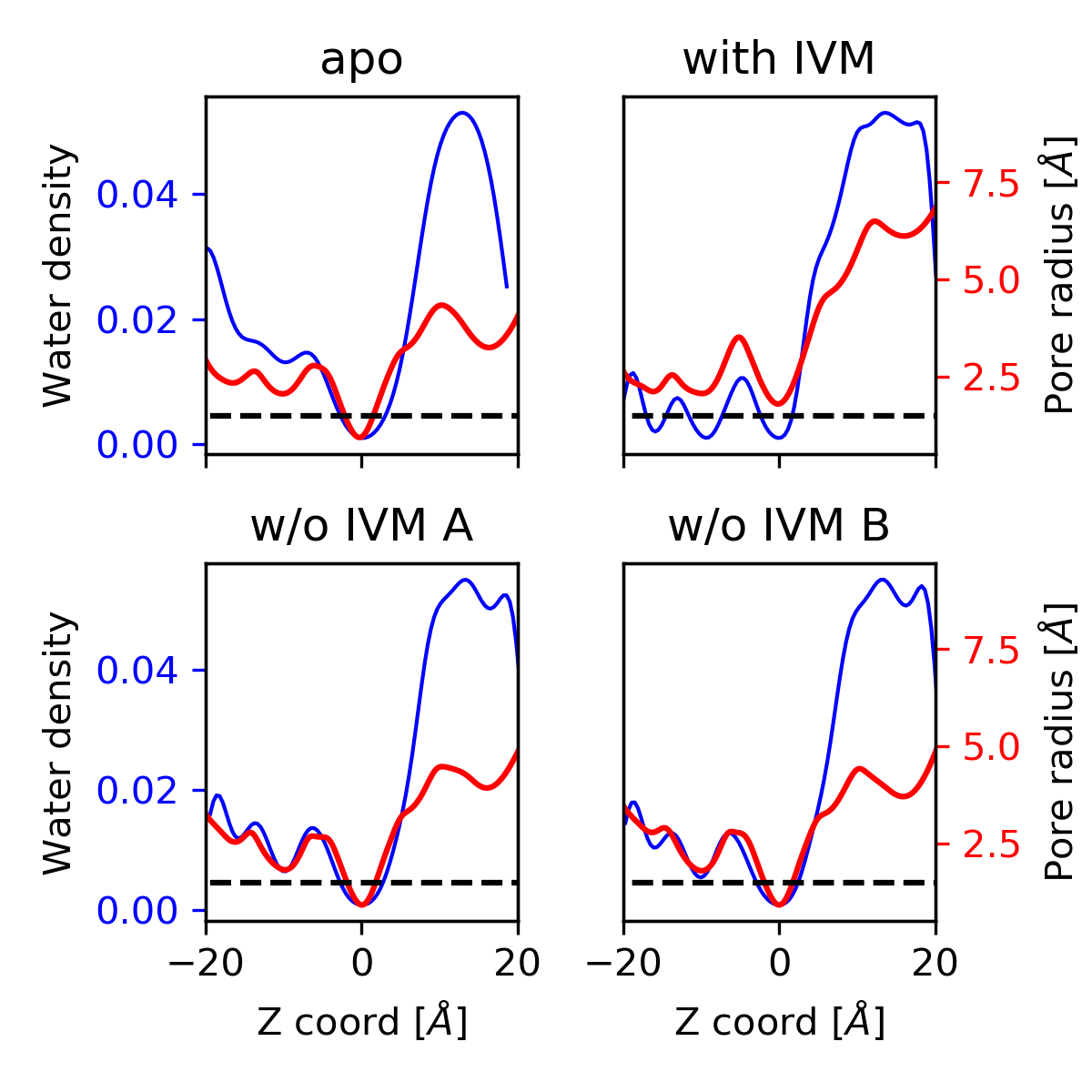

Supplement: S17 Fig — The zero of the Z coord corresponds to the position of residues at 9′. The dashed black line represents the radius of a water molecule (1.5 Å). The dehydration stretch is measured as the extension of the pore region where the water density is below one hundredth of the bulk. The length of the dehydrated stretch is 8.4, 4.8 and 4.6 Å in apo, w/o IVM run A and run B, respectively. (TIF) [file pcbi.1005784.s022.tif]
